# Underestimation of Thermogenic Methane Emissions in New York City

**DOI:** 10.1021/acs.est.3c10307

**Published:** 2024-05-14

**Authors:** Joseph R. Pitt, Israel Lopez-Coto, Anna Karion, Kristian D. Hajny, Jay Tomlin, Robert Kaeser, Thilina Jayarathne, Brian H. Stirm, Cody R. Floerchinger, Christopher P. Loughner, Róisín Commane, Conor K. Gately, Lucy R. Hutyra, Kevin R. Gurney, Geoffrey S. Roest, Jianming Liang, Sharon Gourdji, Kimberly L. Mueller, James R. Whetstone, Paul B. Shepson

**Affiliations:** † School of Marine and Atmospheric Sciences, Stony Brook University, Stony Brook, New York 11794, United States; ‡ National Institute of Standards and Technology, Gaithersburg, Maryland 20899, United States; § Department of Chemistry, Purdue University, West Lafayette, Indiana 47907, United States; ∥ School of Aviation and Transportation Technology, Purdue University, West Lafayette, Indiana 47906, United States; ⊥ Department of Earth and Planetary Sciences, Harvard University, Cambridge, Massachusetts 02138, United States; # Air Resources Laboratory, NOAA, College Park, Maryland 20740, United States; ¶ Department of Earth and Environmental Sciences, Lamont-Doherty Earth Observatory, Columbia University, Palisades, New York 10964, United States; ∇ Department of Earth and Environment, Boston University, Boston, Massachusetts 02215, United States; ○ School of Informatics, Computing and Cyber Systems, Northern Arizona University, Flagstaff, Arizona 86011, United States; ⧫ Environmental Systems Research Institute, Redlands, California 92373, United States

**Keywords:** urban emissions, greenhouse gas emissions, methane, airborne greenhouse gas measurements, Bayesian inverse modeling, emissions inventory development, New York City

## Abstract

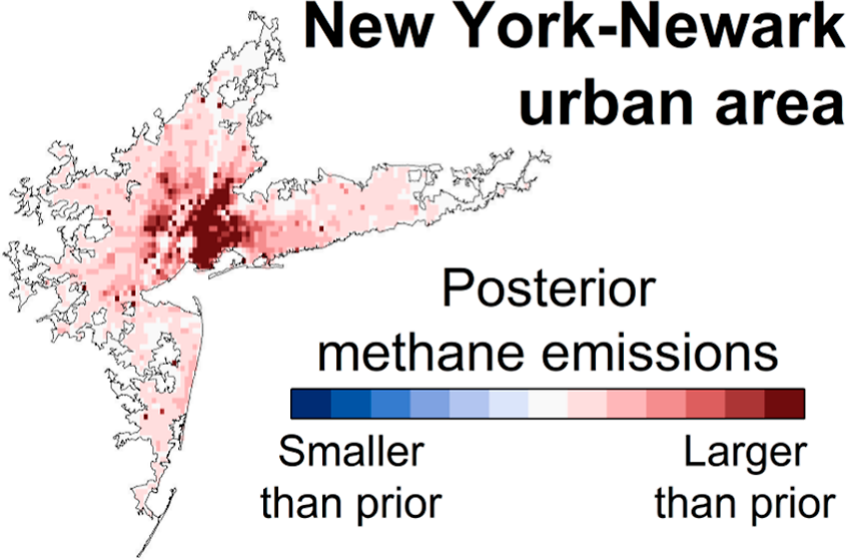

Recent studies have shown that methane emissions are underestimated by inventories in many US urban areas. This has important implications for climate change mitigation policy at the city, state, and national levels. Uncertainty in both the spatial distribution and sectoral allocation of urban emissions can limit the ability of policy makers to develop appropriately focused emission reduction strategies. Top-down emission estimates based on atmospheric greenhouse gas measurements can help to improve inventories and inform policy decisions. This study presents a new high-resolution (0.02 × 0.02°) methane emission inventory for New York City and its surrounding area, constructed using the latest activity data, emission factors, and spatial proxies. The new high-resolution inventory estimates of methane emissions for the New York-Newark urban area are 1.3 times larger than those for the gridded Environmental Protection Agency inventory. We used aircraft mole fraction measurements from nine research flights to optimize the high-resolution inventory emissions within a Bayesian inversion. These sectorally optimized emissions show that the high-resolution inventory still significantly underestimates methane emissions within the New York-Newark urban area, primarily because it underestimates emissions from thermogenic sources (by a factor of 2.3). This suggests that there remains a gap in our process-based understanding of urban methane emissions.

## Abstract 2

Inventories typically underestimate methane emissions in US cities, impeding emission reduction efforts. This study highlights underestimation of thermogenic methane emissions in New York City, by combining aircraft-based measurements with a newly developed inventory.

## Introduction

It is essential to reduce anthropogenic methane emissions to mitigate climate change. A recent report by the United Nations Environment Programme concluded that “reducing human-caused methane emissions is one of the most cost-effective strategies to rapidly reduce the rate of warming and contribute significantly to global efforts to limit temperature rise to 1.5 °C.”^1^ This is because warming due to short-lived climate pollutants such as methane is largely dependent on the current rate of emission, in contrast to gases such as carbon dioxide, for which induced warming depends on cumulative past emissions.^2^ Over 100 countries have now signed the Global Methane Pledge,^3^ committing to contribute toward a 30% reduction in global anthropogenic methane emissions by 2030, relative to 2020 levels.

Urban areas are large sources of anthropogenic methane emissions, but recent studies have shown that methane emissions are significantly underestimated by emission inventories in several US cities.^4−11^ Top-down studies (based on atmospheric measurements of methane enhancements) have estimated methane emissions from the census-designated New York-Newark urban area^12^ (hereafter referred to as the NY-UA) that are over a factor of 2 higher than the gridded Environmental Protection Agency (GEPA) inventory.^5,6^ Plant et al.^5^ combined aircraft measurements of the CH_4_/CO_2_ enhancement ratio with inventory CO_2_ data to estimate an NY-UA emission rate 2.7 times larger than the GEPA. Pitt et al.^6^ used aircraft CH_4_ measurements in an inverse modeling framework to derive a posterior estimate for NY-UA methane emissions that was 2.4 times larger than the GEPA.

Inventory underestimation of thermogenic (i.e., fossil fuel) methane sources has been identified as an important factor contributing toward the observed discrepancy with top-down studies.^5,13^ The GEPA inventory was compiled by spatially disaggregating the US national total methane emissions for the year 2012, as reported in the 2016 EPA national inventory report (NIR), using a range of spatial proxies.^14^ It is therefore not representative of emissions in more recent years, and it does not incorporate more recent studies that have provided updated emission factors for key sources (e.g., natural gas emissions from distribution pipelines).^15^ Furthermore, the GEPA inventory was designed for studies assessing national and regional scale emissions and has a spatial resolution of 0.1 × 0.1°. For accurate assessment and modeling of urban emissions, we need information at a higher spatial resolution.

In this study, we present a new high-resolution (0.02 × 0.02°) inventory of 2019 methane emissions focused on the NY-UA and its surroundings, with the objective of disentangling thermogenic and nonthermogenic emissions, and thereby calculating the thermogenic fraction of emissions (i.e., the fraction of emissions from thermogenic sources). We use data from nine research aircraft flights to sectorally optimize the high-resolution inventory emissions, deriving separate posterior estimates of thermogenic and nonthermogenic emissions by leveraging the different spatial distributions of emissions from these sectors. This approach differs from previous top-down studies that have relied on measurements of CH_4_ isotopic composition or coemitted tracers such as ethane (C_2_H_6_) to estimate the relative contribution from thermogenic emission sources.^4,5,8,13,16−20^ It relies on the identification of distinctly different spatial emission patterns for these sectors, which is enabled through the compilation of a high-resolution inventory. We then benchmark this new sectorally optimized inversion against total emission estimates derived using the well-established spatial-optimization inverse modeling approach presented by Pitt et al.^6^ Through this analysis, we are able to

1. Estimate thermogenic and nonthermogenic emissions for the NY-UA using a bottom-up inventory method based on the latest emission factors and activity data.
2. Optimize these thermogenic and nonthermogenic emission estimates using aircraft data.
3. Assess the inventory based on these optimized emissions and identify key sectors that should be the focus for future improvement.

## Methods

### High-Resolution Inventory

The construction of a high-resolution inventory extended the approach used by McKain et al.^8^ and Sargent et al.^4^ With reference to the GEPA and the literature, we identified sources for which significant CH_4_ emissions could be expected in the NY-UA and its surrounding areas. We calculated emissions from these sectors using activity data for the year 2019, when most of our research aircraft flights were conducted. We calculated emission totals for each sector by multiplying these activity data by emission factors taken from the latest literature (see Supporting Information Tables S1.2–S1.5 for values). We used different spatial proxies for each sector (detailed in the subsections below) to obtain an emissions map on a 0.02 × 0.02° grid, over a domain bounded by 39.2° N, 42.0° N, 75.7° W, and 72.1° W. This domain encompasses all or part of five states: New York (NY), Connecticut (CT), New Jersey (NJ), Pennsylvania (PA), and Delaware (DE). Here we describe how the emission map was constructed for each of the seven main sectors. In some cases, we created multiple maps for the same sector to reflect uncertainty in the underlying assumptions.

#### Landfills

This sector includes municipal and industrial landfills. Where municipal solid waste landfills reported emissions for the year 2019 to the EPA Greenhouse Gas Reporting Program (GHGRP),^21^ we used these GHGRP-reported values. For landfills in the Landfill Methane Outreach Program database^22^ that did not report to the GHGRP (including closed landfills), we calculated a default emission rate, unless they were labeled “facility discontinued reporting without a valid reason” in the GHGRP, in which case we used their most recent GHGRP-reported value. We calculated a default emission rate of 0.52 mol s^–1^ landfill^–1^ by evenly distributing non-GHGRP emissions from the EPA NIR.^23^ We took emissions for industrial solid waste landfills directly from the GEPA inventory at its native 0.1° resolution.

#### Wetlands and Inland Waters

This sector includes wetlands, lakes, and rivers. We constructed three different emission maps for the wetland fluxes. The first was based on WetCHARTs v1.3.1,^24^ using cold-season values, spatially downscaled based on land cover from the National Land Cover Database (NLCD, 2016).^25^ The other two used the wetland fraction from the National Wetlands Inventory (NWI),^26^ with wetland fluxes taken from either the First State of the Carbon Cycle Report (SOCCR1)^27^ or the Second State of the Carbon Cycle Report (SOCCR2).^28,29^ We took locations of rivers and lakes from the NWI and assigned them fluxes taken from Rosentreter et al.^30^ (see Supporting Information Section S1 for details).

#### Natural Gas Distribution

This sector contains emissions from the following natural gas distribution subsectors: mains pipelines, service pipelines, consumer meters, maintenance/upsets, and M&R (metering and regulating) stations. For each subsector, we calculated emissions at either the local distribution company (LDC) level, the state level, or the five-state level (i.e., the total for all five states that intersect our domain: NY, CT, NJ, PA, DE). Within these areas, we spatially distributed emissions proportional to reported carbon dioxide (CO_2_) emissions from residential and commercial fossil fuel combustion, taken from either the Anthropogenic Carbon Emissions System (ACES) version 2.0 inventory^31,32^ or the Vulcan version 3.0 inventory.^33,34^ We allocated consumer meter emissions from residential and commercial meters to the corresponding ACES/Vulcan map. For other subsectors, we used the weighted average of the ACES/Vulcan residential and commercial CO_2_ maps, with the weights given by the number of residential and commercial natural gas customers, respectively.

We used LDC service territory shapefiles from the Homeland Infrastructure Foundation-Level (HIFLD) database.^35^ Some companies could not be matched to a polygon in this database. We aggregated these companies into a single auxiliary company for each state, whose polygon covered all areas not covered by a service territory in HIFLD. Emissions from these companies represented 6.4% of the total emissions for the five states.

We calculated total emissions from mains pipelines using the miles of main reported in the Pipelines and Hazardous Materials Safety Administration (PHMSA) database for 2019,^36^ broken down by material. We combined these activity data with emission/activity factors from Weller et al.^15^ For all other subsectors, we took emission factors from the EPA NIR.^23^ We calculated service pipeline emissions based on the number of services by material (PHMSA) and emissions from maintenance/upsets based on the total miles of mains and service pipelines (PHMSA). We calculated emissions from consumer meters based on the number of individual consumers reported to the US Energy Information Administration (EIA).^37^ We based emissions from M&R stations on facility counts from the GHGRP and assigned values to nonreporters based on the average number of M&R stations per mile for GHGRP-reporting companies. See Supporting Information Table S1.2 for a summary of emission factors from the various subsectors.

#### Natural Gas Residential Post Meter

This sector contains emissions associated with natural gas leakage downstream of residential meters. We estimated postmeter emissions as 0.5% of total residential consumption, based on Fischer et al.^38^ We took consumption data from EIA reports^37^ at either the LDC level, state level or five-state level. We then distributed emissions within these areas in proportion to reported CO_2_ emissions from residential fossil fuel combustion, taken from either ACES version 2.0 or Vulcan version 3.0.

#### Natural Gas Transmission

This sector contains emissions associated with the natural gas transmission network. We calculated a default emission factor for compressor stations, based on US total emissions and the number of stations reported in the EPA NIR.^23^ For compressor stations that reported to the GHGRP, we assigned emissions proportional to their GHGRP values but rescaled such that the average emission rate per station within our domain was equal to the calculated default emission factor. We used the HIFLD database^39^ to determine the location of stations that did not report to the GHGRP; these stations were all allocated the same default emission value. We took other emissions (not associated with compressor stations) from the EPA NIR^23^ and allocated them uniformly along transmission pipelines (see Supporting Information Section S1 for details).

#### Stationary Combustion of Fossil Fuels and Wood

We estimated emissions from stationary combustion of fossil fuels and wood using fuel- and consumer-type-specific emission factors reported by the Intergovernmental Panel on Climate Change,^40^ except for emissions from natural gas use in the electricity production sector where we used emission factors from Hajny et al.^41^ We excluded residential natural gas emissions here because they were already accounted for in the natural gas residential post meter sector. We used consumption data (in Btu) from the EIA State Energy Consumption Estimates database,^42^ broken down by fuel and consumer type (residential, commercial, industrial, electric power), and applied correction factors, taken from the EPA NIR,^23^ to avoid double counting emissions already reported in other sectors (see Supporting Information Table S1.3).

We calculated either state-total or five-state-total emissions for each fuel and consumer type. We spatially disaggregated these totals to the county level in proportion to fuel- and consumer-type-specific CO emissions from the National Emissions Inventory for the year 2017.^43^ We then spatially distributed these emissions within each county in proportion to reported CO_2_ emissions for the corresponding consumer type, taken from either ACES version 2.0 or Vulcan version 3.0 (these inventories do not contain fuel-specific information). We have grouped emissions associated with fossil fuel combustion in subsequent analysis but kept wood combustion emissions separate.

#### Wastewater

This sector contains emissions from municipal and industrial wastewater treatment plants (WWTPs) as well as onsite treatment (e.g., septic tanks). We calculated emissions from centralized domestic WWTPs by taking the national total from the EPA NIR^23^ and allocating it across all WWTPs that reported to the 2012 Clean Watersheds Needs Survey,^44^ in proportion to their reported existing municipal flow. We took emissions for industrial WWTPs directly from the GHGRP. We estimated emissions from onsite systems by spatially disaggregating either national- or state-total emissions. We took national emissions from the EPA NIR^23^ and calculated state-total emissions by multiplying an estimate for the number of people within each state using onsite systems (see Supporting Information Section S1 for details) by the emission factor from the EPA NIR (10.7 g of methane per capita per day).

We spatially distributed these national- and state-total emissions according to the fraction of each grid cell categorized as “Developed-Open Space” or “Developed-Low Intensity” in the 2016 NLCD.^25^ We based this assumption on the consideration that most onsite treatment systems exist in exurban areas and small towns where these land classes dominate (i.e., places where there are residents but often no sewers).

#### Other

We took other minor emitting sectors directly from the GEPA inventory (see Supporting Information Section S1 for a list of sectors).

### Aircraft Measurements

The nine research aircraft flights used in this study (conducted using the Purdue University Airborne Laboratory for Atmospheric Research) were previously presented by Pitt et al.,^6^ and the aircraft instrumentation for trace gas measurements was described by Cambaliza et al.^45^ We conducted these flights on nine different days between November 2018 and March 2020. We timed the flights to ensure sampling downwind of the urban area occurred between late morning and late afternoon when the boundary layer was relatively well mixed. See Supporting Information Section S3 for more details about these aircraft measurements.

### Transport Modeling

We calculated timeseries of modeled mole fractions based on each version of the high-resolution inventory using HYSPLIT v5.0.0 (Hybrid Single Particle Lagrangian Integrated Trajectory Model).^46−48^ We ran an ensemble of eight HYSPLIT configurations for each flight, based on input meteorology from four different sources (the European Centre for Medium Range Weather Forecasts Fifth Reanalysis, ERA5; the NOAA Global Forecast System model, GFS; the NOAA North American Mesoscale Forecast System model, NAM; and the NOAA High-Resolution Rapid Refresh model, HRRR) and two different turbulence parametrizations (Hanna^49^ and Kantha–Clayson^50^). Full details of the HYSPLIT ensemble runs are provided by Pitt et al.^6^ (including example HYSPLIT configuration files in the Supporting Information File accompanying that study). We performed a separate HYSPLIT run for every minute of each flight that contained sampling within the boundary layer and evenly distributed the HYPLIT model particle release over all aircraft sampling locations within that minute. HYSPLIT tracks the trajectory of these particles backward in time, enabling us to calculate a modeled time series representing the influence of surface fluxes on the sampled air. Note that we used all of the inventory sectors as tagged tracers in each of the transport models.

### Inverse Modeling

We used two different inverse modeling approaches in this study: a “spatial” approach and a “sectoral” approach. In both cases, we optimized fluxes separately for each flight. The spatial approach replicated the inverse modeling framework applied by Pitt et al.^6^ Here, we optimized fluxes at the grid-cell level, using a nested inversion approach that first optimized fluxes on a large, coarse (0.08 × 0.08°) domain (bounded by 34.4° N, 44.4° N, 83.7° W, 69.7° W), hereafter referred to as the d01 domain. For the parts of d01 not covered by the high-resolution inventory, we used prior anthropogenic emissions from the GEPA inventory. See Supporting Information Section S1 for a description of prior d01 emissions from natural sources.

We used the posterior fluxes from the d01 inversion to provide boundary conditions that enabled the optimization of fluxes on a smaller high-resolution (0.02 × 0.02°) domain, corresponding to the domain of the high-resolution inventory (bounded by 39.2° N, 42.0° N, 75.7° W, 72.1° W), hereafter referred to as the d03 domain (see Supporting Information Section S4 for a map of the nested domains). Note that the prior CH_4_ flux maps used by Pitt et al.^6^ in the original study were natively coarse (0.1 × 0.1°), so while the posterior fluxes did show spatial variability at scales finer than the native prior resolution, they were nevertheless partially constrained by it. Repeating this inversion approach with the high-resolution inventory facilitated a direct comparison of posterior fluxes with previous results.

The sectoral inverse modeling approach used in this study aimed to exploit the higher native resolution of the high-resolution inventory to retain sectoral emissions information within the posterior fluxes. In this case, the modeled mole fractions were split into three components:

1. Emissions outside the NY-UA (within both the d03 and d01 domains).
2. Thermogenic emissions from within the NY-UA.
3. Nonthermogenic emissions from within the NY-UA.

We optimized scaling factors corresponding to each of these components so as to reduce the model-measurement mismatch, subject to prior constraints, by minimizing the cost function
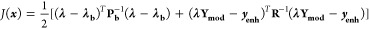
1

Here, **λ** is a vector consisting of three scaling factors that are applied to the three model components, **λ**_**b**_ is the prior scaling factor estimate, set to 1 for all sectors, **P**_**b**_ is the prior error covariance matrix, **Y**_**mod**_ is a matrix containing timeseries for the three model components, ***y***_**enh**_ is a vector containing the measured enhancements (above background—see Supporting Information Section S4 for details), and **R** is the model-measurement mismatch error covariance matrix, derived for each sample period (1 min) from the measurement variability, the variability across the HYSPLIT model ensemble, and the background uncertainty (as described by Pitt et al.).^6^

We set the prior uncertainty () to 50% for all three components based on the aggregate uncertainty for the urban area of an “equivalent” spatial inversion. Since in the urban area, we optimize two uncorrelated sectors, each with a 50% relative uncertainty, the total relative uncertainty for the urban area would be 70%. This is the same as a spatial inversion with a 70% relative uncertainty for each grid cell and perfect correlation among the grid cells. Similar total uncertainty would result from the use of 240% per-pixel uncertainty and 10 km correlation length or 85% per-pixel uncertainty and 100 km correlation length. As a sensitivity test, we repeated the optimization with  set to 25 and 100% (see Supporting Information Section S5 for more details). In contrast to the spatial inversion and Pitt et al.,^6^ we set the nondiagonal terms in the error covariance matrices to zero. We adopted this approach because scaling multiple grid cells with the same scaling factor implies a correlation of 1 between those grid cells; thus, incorporating additional correlations was deemed unnecessary.

Once the three posterior scaling factors were calculated, we derived separate posterior flux maps for the thermogenic and nonthermogenic sources within the NY-UA by multiplying the prior fluxes for these sectors by the corresponding posterior scaling factor. This approach leverages the contrasting spatial patterns exhibited by emissions from these sectors to derive separate posterior scaling factors. Further disaggregation to optimize individual thermogenic and nonthermogenic sectors was not possible because the spatial distribution of the sectors was less distinct and small sectors had too little signal to be confidently separated. An approach following a similar principle was recently applied to satellite data from NASA’s Orbiting Carbon Observatory-3 to estimate sector-specific CO_2_ fluxes for the Los Angeles Basin.^51^

## Results and Discussion

### High-Resolution Inventory

The development of multiple possible flux maps (i.e., inventory versions) for some sectors resulted in many possible versions of the new high-resolution inventory. We initially produced 144 versions of the inventory, but the remainder of the analysis focuses on 4 selected versions, hereafter labeled HRA, HRB, HRC, and HRD. We chose these versions following an analysis of the correlation between modeled and measured timeseries, detailed in Supporting Information Section S2. We selected the versions with the highest mean and median *r*^2^ values (HRA and HRC) as well as two other versions that calculated sector totals for the smallest possible area before disaggregating (e.g., using LDC-level emissions to calculate natural gas distribution/postmeter emissions) had high median *r*^2^ values and used ACES (HRB) and Vulcan (HRD), respectively, as spatial proxies. The differences between these versions are outlined in Table 1.

**Table 1:** Four Versions of the High-Resolution Inventory That Form the Focus of This Study

version	natural gas distribution/postmeter		stationary combustion (fossil fuels and wood)		wetlands		onsite wastewater treatment
	emission level	spatial proxy	emission level	spatial proxy	emission factor	spatial map	emission level
HRA	state	ACES	state	Vulcan	WetCHARTs	NLCD	national
HRB	LDC	ACES	state	ACES	WetCHARTs	NLCD	state
HRC	5-state	Vulcan	state	Vulcan	SOCCR1	NWI	state
HRD	LDC	Vulcan	state	Vulcan	SOCCR1	NWI	state

**Figure 1:**
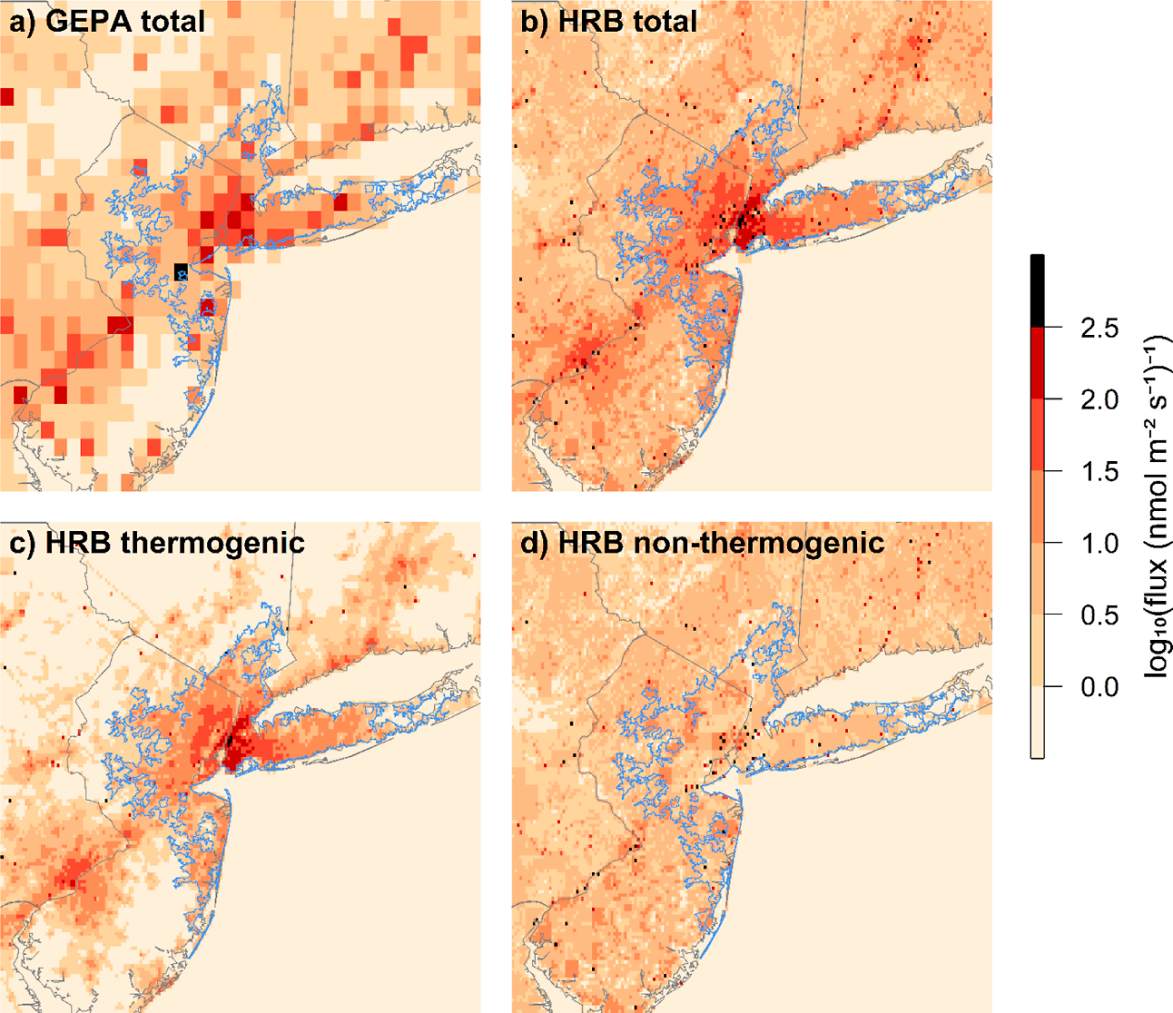
Panel plot showing flux maps for (a) GEPA (all sectors), (b) high-resolution inventory (all sectors), (c) high-resolution inventory (thermogenic sectors), and (d) high-resolution inventory (nonthermogenic sectors). All plots show fluxes on a logarithmic scale. The NY-UA outline is shown in blue. The high-resolution inventory version shown here is version HRB.

Figure 1 shows flux maps for the GEPA and the high-resolution inventory (version HRB). These highlight the fine-scale structure in CH_4_ fluxes that is not captured by 0.1° resolution inventories. The thermogenic and nonthermogenic flux maps for the high-resolution inventory are also shown separately. Thermogenic emissions are predominantly located in large population centers, with some point source emissions from natural gas transmission compressor stations. Nonthermogenic emissions are dominated by point sources and small-area sources with known locations, such as landfills and municipal WWTPs (many of which serve over a million people), with a much smaller diffuse emission pattern that includes natural emissions and onsite wastewater treatment emissions (e.g., septic tanks).

**Figure 2:**
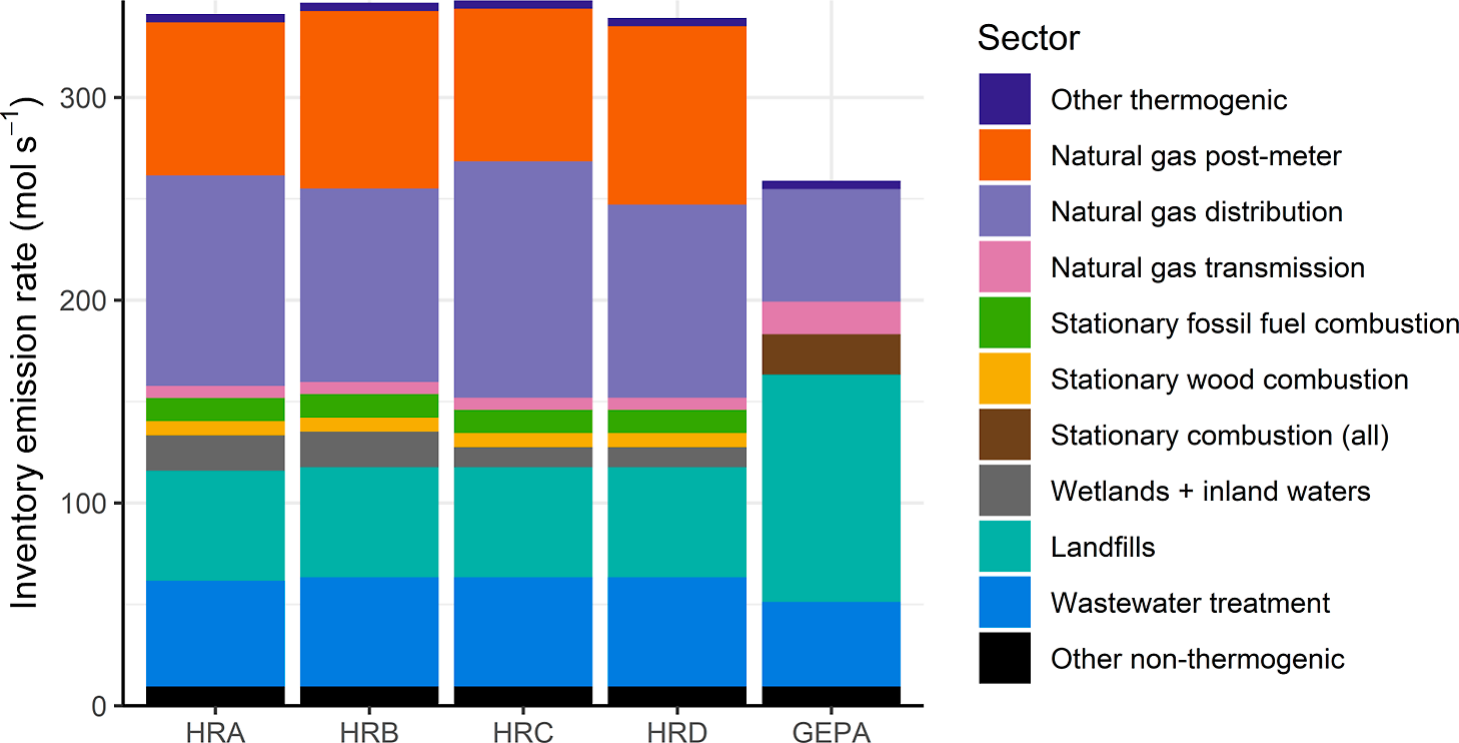
Stacked bar chart showing prior emission rates for the NY-UA from four versions of the high-resolution inventory and the GEPA.

Figure 2 shows a sectoral breakdown of NY-UA emissions from the four selected versions of the high-resolution inventory (these data are also provided in Supporting Information Table S5.1). The fraction of total emissions from thermogenic sources is considerably larger in all four versions of the high-resolution inventory (approximately 0.6) compared to the GEPA (0.37). Emissions from the natural gas distribution system are larger in the high-resolution inventory than the GEPA (by at least 70% in these four cases), primarily as a result of using the higher activity factors (leaks per mile) and emission factors (emissions per leak) reported by Weller et al.^15^ The inclusion of postmeter residential natural gas emissions in the high-resolution inventory also results in a substantial increase in estimated thermogenic emissions. It is worth noting that while postmeter emissions are not included in the GEPA, the 2022 EPA NIR^52^ does (for the first time) include emissions from this sector.

Smaller landfill emissions in the high-resolution inventory relative to the GEPA, as a consequence of a decline in GHGRP-reported emissions between 2012 and 2019, also contribute to the increase in the thermogenic fraction. It is worth noting that some landfills changed their GHGRP reporting methodology between 2012 and 2019 (the relevant federal regulations specify two alternative calculation methods: equations HH-6 and HH-8),^53^ which could have contributed to this decline in reported emissions.

The new high-resolution inventory yields an emissions total for the NY-UA that is 1.3 times larger than in the GEPA (using any of the four high-resolution inventory versions). While this brings the high-resolution inventory closer to the total NY-UA emission rates reported by Plant et al.^5^ and Pitt et al.,^6^ it is still much lower than the mean scaling factors of 2.7 and 2.4 they, respectively, estimate. This suggests that there are still emission sources that are either underestimated by or missing from our high-resolution inventory.

### Spatial Inversion

The mean posterior NY-UA emission rate from the spatial inversion was (585 ± 171) mol s^–1^ [(9.39 ± 2.75) kg s^–1^], where the uncertainty reported throughout refers to the 1σ temporal variability of the ensemble-average results across the nine flights (29%). This is similar to the mean posterior result of (616 ± 188) mol s^–1^ reported by Pitt et al.^6^ using the same aircraft measurement data set and HYSPLIT model runs, but a different set of prior flux maps.

While Pitt et al.^6^ found that the magnitude of prior emissions had a non-negligible impact on posterior emissions, the close agreement between the results of these two studies gives us confidence that the ensemble approach used by Pitt et al.^6^ was able to mitigate the prior dependency and that these posterior emission rate estimates are not overly sensitive to the precise spatial distribution of the prior (including its spatial resolution). The mean posterior emission rate from the spatial inversion is 1.7 times larger than the prior emission rates in the high-resolution inventory and 2.3 times larger than that from the GEPA (as shown in Figure S5.3). See Supporting Information Figure S5.4 for a detailed breakdown of results from the spatial inversion.

### Sectoral Inversion

#### Total Emissions

**Figure 3:**
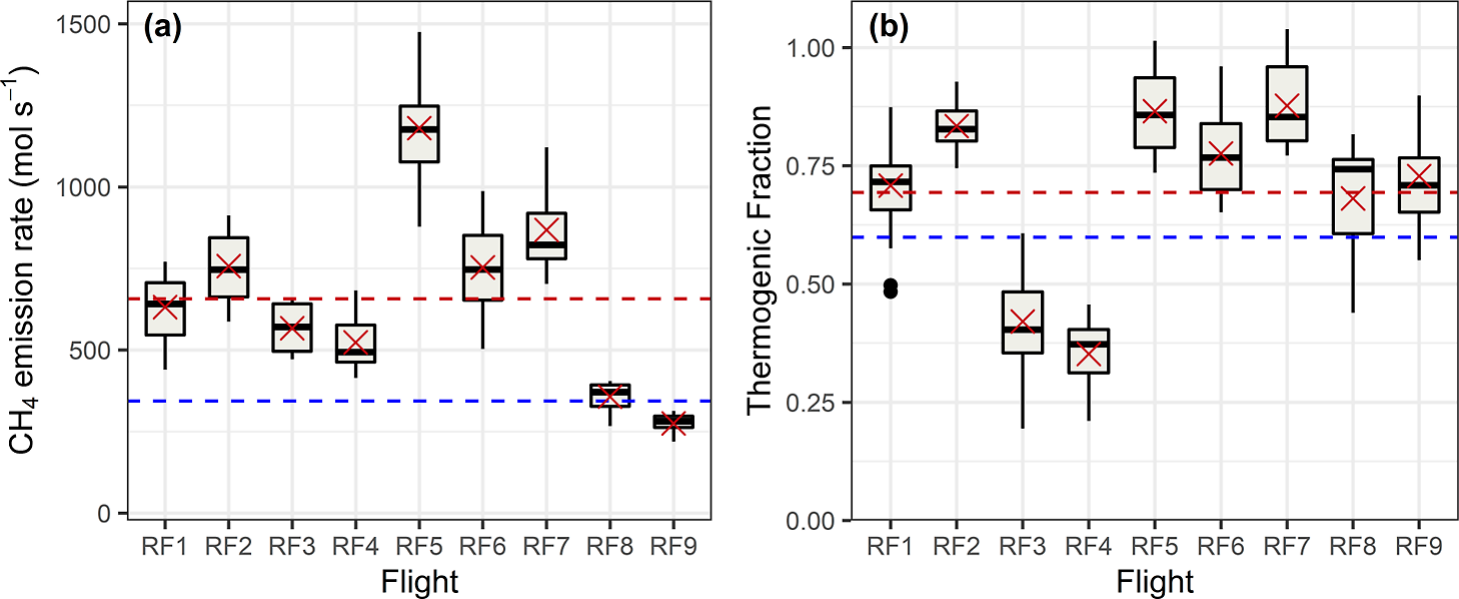
Posterior sectoral inversion results for the NY-UA showing the (a) total emission rate and (b) thermogenic fraction. A separate boxplot is shown for each flight, comprised of results derived using the four priors and eight transport models. Mean posterior results for each flight are shown as red crosses, with the overall mean posterior values shown as red dashed lines. The mean prior values (averaged over the four priors) are shown as blue dashed lines. See Supporting Information Figure S5.4 caption for boxplot convention.

Figure 3 shows the results of the sectoral inversion (with a more detailed breakdown in Supporting Information Figure S5.5). The mean posterior total emission rate from the sectoral inversion was (657 ± 274) mol s^–1^ [(10.5 ± 4.4) kg s^–1^]. This total emission rate is 1.12 times larger than the corresponding value derived using the spatial inversion, 1.07 times larger than Pitt et al.,^6^ 1.9 times larger than the high-resolution inventory prior, and 2.5 times larger than the GEPA. The flight-to-flight variability of the posterior total emission rate is larger for the sectoral inversion (42%) than the spatial inversion (29%), showing that the prior constraint on total emissions is weaker in the case of the sectoral inversion (this can also be seen by comparing Supporting Information Figures S5.4 and S5.5).

#### Thermogenic Fraction

The sectoral inversion provides information about the posterior thermogenic fraction that is not provided by the spatial inversion. The posterior thermogenic emission rate was, on average, 2.3 times larger than the corresponding prior thermogenic emission rate. The average posterior nonthermogenic emission rate was also larger than the prior, but by a smaller factor of 1.3. The mean posterior thermogenic fraction was 0.69 (taken as an average over the individual thermogenic fractions calculated for each flight and model ensemble member), with a 1σ flight-to-flight variability of 0.19 (27%). This mean posterior thermogenic fraction is larger than the prior thermogenic fractions for all four inventory versions (approximately 0.6), as shown in Supporting Information Figure S5.3. We note that we get a slightly higher result of 0.73 if we divide the mean posterior thermogenic emissions by the mean posterior total emissions (because the mean of fractions is not equal to the fraction of the means).

#### Comparison with Previous Studies

It is clear from these results that the inventory significantly underestimates the NY-UA CH_4_ emissions sampled by our flights and that the key underestimated, or missing, sources are likely related to thermogenic sectors. High-resolution inventories have also been compiled using similar methods for the Boston^4,8^ and Washington, DC–Baltimore^54^ urban areas. In Boston, ground-based measurements of CH_4_ and C_2_H_6_ suggested that the natural gas loss rate was three times higher than the 0.8% loss rate in the inventory.^4^ Posterior emission totals for Washington, DC, and Baltimore were in relatively good agreement with inventory estimates that included a 1.5% natural gas loss rate in addition to bottom-up estimates of emissions from natural gas distribution and transmission.^54^ Our results are therefore in line with previous reports that thermogenic methane emissions are typically underestimated by bottom-up methods in urban areas throughout the US.^4^

It is important to note that the inventory is designed to represent annual emissions for 2019, so it does not reflect the exact dates and times of the emissions sampled by our flights. Diurnal, weekly ,and seasonal variability in CH_4_ emissions may be expected for some sources, related to meteorological conditions (e.g., landfill) and certain human activities (e.g., incomplete combustion from household appliances), as demonstrated by seasonal variability reported previously in Los Angeles^11^ and Washington, DC–Baltimore.^9,54^

It is not possible to detect seasonal or diurnal patterns in CH_4_ emissions for the NY-UA using the nine flights presented in this study. However, our mean posterior thermogenic fraction of 0.69 ± 0.19 is consistent with top-down thermogenic fraction estimates based on aircraft measurements of C_2_H_6_/CH_4_ enhancement ratios. Plant et al.^5^ estimated a thermogenic fraction of 0.87_–0.12_^+0.10^ for the NY-UA (95% confidence interval) based on C_2_H_6_/CH_4_ data from ten flight days in April and May 2018. Floerchinger et al.^13^ also used C_2_H_6_/CH_4_ data to derive New York City thermogenic fractions of 0.815_–0.006_^+0.022^ and 1.188_–0.028_^+0.022^ for individual flights in September 2017 and March 2018 respectively (95% confidence interval), with the latter value potentially influenced by entrainment of free tropospheric air (or other issues). Relative to these previous studies, the mean posterior thermogenic fraction from our sectoral inversion implies a larger role for nonthermogenic sectors in the NY-UA source mix, suggesting that they should not be disregarded as negligible. However, there is substantial overlap between our 1σ flight-to-flight variability and the 95% confidence intervals reported by Plant et al.^5^ and by Floerchinger et al.^13^ for their September flight. All three studies estimate larger thermogenic fractions than both the GEPA (0.37) and the four versions of the high-resolution inventory (approximately 0.6), despite using different estimation techniques and covering different months of the year. Coupled with the fact that top-down estimates of total emissions from both this study and Plant et al.^5^ are much larger than those estimated by the high-resolution inventory, it can be considered likely that the high-resolution inventory underestimates thermogenic CH_4_ emissions at the annual scale.

The sectoral inversion approach adopted here relies on distinct spatial distributions for thermogenic and nonthermogenic sources. Figure 1 shows that our high-resolution inventory exhibits such distinct spatial patterns. However, a recent study in Montreal^55^ found emissions from sewers were larger in total than emissions from natural gas distribution infrastructure. To estimate the likely magnitude of sewer emissions in the NY-UA (which are not included in our inventory), we used road-length data from the US Census Bureau^56^ in combination with emission factors (per km of surveyed road) reported for Paris (0.036 t km^–1^ a^–1^),^57^ Utrecht (0.053 t km^–1^ a^–1^), and Hamburg (0.019 t km^–1^ a^–1^).^18^ Based on these values, we estimate NY-UA sewer emissions that are between 1.0 and 2.7% of total NY-UA inventory emissions. A much larger emission factor (0.38 t km^–1^ a^–1^) reported for Bucharest^19^ would yield NY-UA sewer emissions that are equivalent to 19% of total inventory emissions. However, the C_2_H_6_/CH_4_ ratio reported by Plant et al.^5^ and Floerchinger et al.^13^ imply that this value is not representative of sewers in the NY-UA. We therefore conclude that the posterior thermogenic fraction from our sectoral inversion is unlikely to be strongly impacted by sewer emissions, although it is worth noting that sewer emissions can be impacted by meteorology and are unlikely to be constant in time.

#### Variability across Flights and Priors

All four of the high-resolution inventory priors produced very similar posterior results. Campaign-average posterior results showed 1σ variabilities across the priors of 3.9% in total emissions and 2.1% in thermogenic fraction (relative to the mean posterior values). For a single flight, average 1σ variabilities across the priors were 4.0% for both total emissions and the thermogenic fraction. As discussed by Pitt et al.,^6^ the large flight-to-flight variability in the posterior results presented here (42% for total emissions and 27% for thermogenic fraction) likely results from a combination of: (1) real changes in NY-UA emissions between the different flight days, (2) spatiotemporal aliasing of temporally varying emissions due to different sampling patterns (related to different meteorology—see Supporting Information Figure S3.1) for different flights (as described by Lopez-Coto et al.),^7^ and (3) methodological uncertainties (a sensitivity test to assess the impact of the prescribed prior uncertainty is presented in Supporting Information Section S5).

#### Comparing the Sectoral and Spatial Inversions

**Figure 4:**
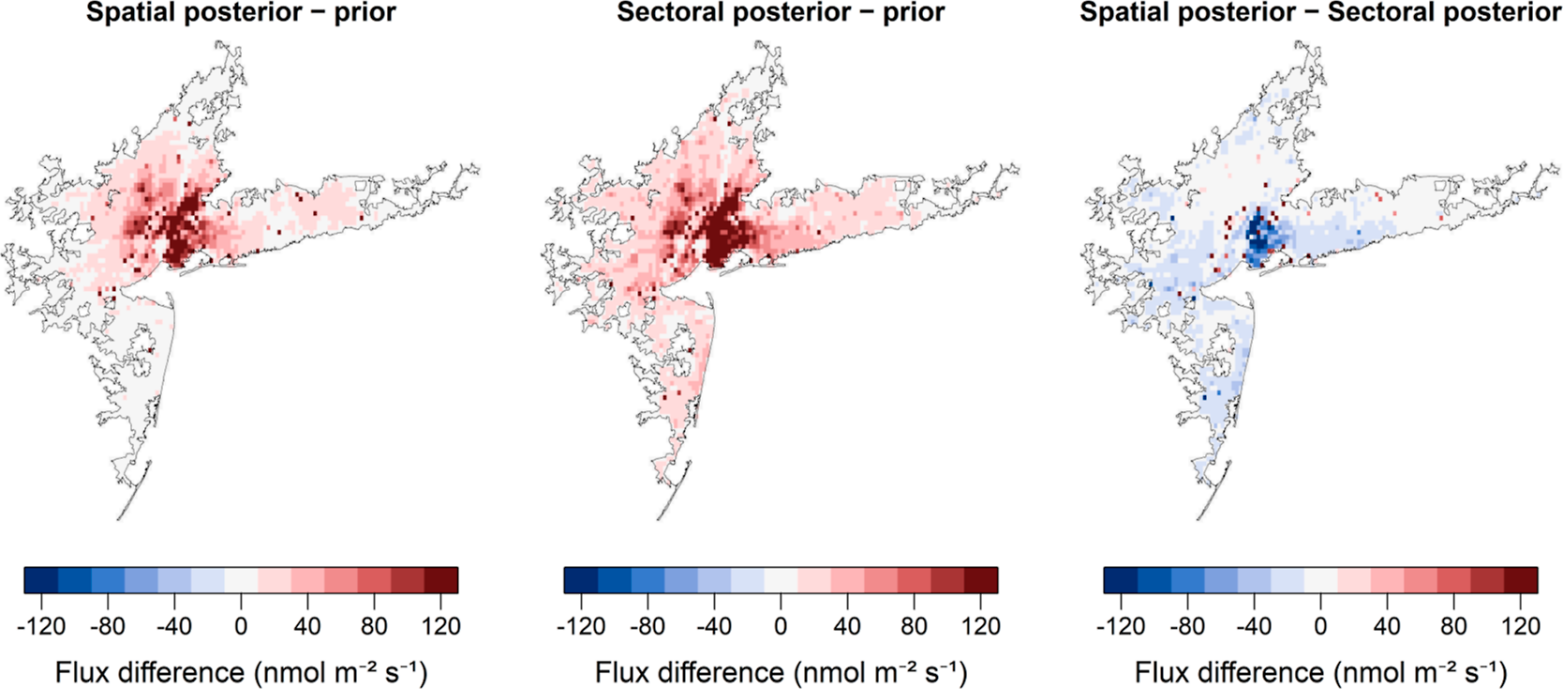
Urban area maps showing the difference between mean posterior and prior fluxes for both the spatial and sectoral inversions (averaged over all flights and models) as well as the difference between the spatial inversion posterior and the sectoral inversion posterior (all using high-resolution inventory version HRB).

Figure 4 shows the spatial distribution of posterior–prior differences for both the spatial and sectoral inversions as well as the difference between posterior flux maps calculated using the two different inversion methodologies. Cells dominated by diffuse thermogenic emissions were upscaled by a larger factor in the sectoral posterior than in the spatial posterior. This is particularly notable within the urban core (Manhattan and Brooklyn), although it is important to emphasize that both inversions yielded larger posterior emissions relative to prior emissions in these areas. Grid cells dominated by nonthermogenic point-source emissions were virtually all higher in the spatial posterior than the sectoral posterior. These patterns follow from the fact that the thermogenic sectors and nonthermogenic sectors were scaled by factors of 2.3 and 1.3, respectively, in the sectoral inversion, while the spatial inversion corrected emissions in each grid cell regardless of their sectoral breakdown. It is also worth noting that, on-average, sources outside the NY-UA were also scaled up in both the spatial and sectoral inversions, as found in the spatial inversion results of Pitt et al.^6^

The posterior emission estimates derived using the sectoral and spatial inverse modeling approaches represent the most likely solutions under two different sets of assumptions, described in the Methods section. The impact of these differences is presented in more detail in Supporting Information Section S5. However, in this case, the mean posterior NY-UA emission rates derived using the sectoral and spatial inversions agree within 12%, which is well within the flight-to-flight variability for either method. Both results also agree closely with the posterior emission rate estimates of Pitt et al.^6^ The conclusion that NY-UA emissions are significantly underestimated in both the high-resolution inventory and the GEPA is therefore not dependent on the choice of inverse modeling approach.

### Wider Context

The results of this study add to mounting evidence that urban thermogenic methane emissions in the US are underestimated by inventories.^4−11^ Even though we used the latest published emission factors and activity data when compiling our high-resolution inventory, there remains a large gap between our inventory estimate and top-down estimates for NY-UA CH_4_ emissions (as observed in Boston^4^). The results of our sectoral inversion indicate that for this urban area, the majority of missing or underestimated emissions in the inventory are from thermogenic sources. It is nevertheless important to note that many nonthermogenic emission sources are also highly uncertain and cannot be disregarded.

Policy makers need granular information about the emission source mix to develop appropriate mitigation strategies. This information is best provided by inventories, but for urban CH_4_, it is clear that further work is required to improve the accuracy of total emissions estimates and the relative contribution from different sectors. More comprehensive process-based studies are required in order to improve our understanding of the key emission sources. For example, estimates of whole-building natural gas leak rates for the types of residential and commercial buildings typical within the NY-UA would be of particular value.

There is strong evidence for inventory underestimation of urban thermogenic methane emissions in many US cities,^4−11^ while results from Canada^55,58^ and Europe^16,18−20,57,59^ present more of a mixed picture. For most other regions of the world, there is very little top-down information regarding urban methane emissions. In many of these urban areas, a lack of publicly available activity data and representative emission factors presents challenges for the development of a high-resolution methane inventory. The best inventory compilation approach will, therefore, be specific to individual countries and cities. Where high-resolution inventories can be compiled, this study has demonstrated their value, especially when they can be combined with aircraft measurements (or other measurements with appropriate spatial coverage) to yield optimized sectoral emissions.

Including ethane and methane isotopologue measurements on future flights would allow for an extension of this approach by placing additional constraints on the posterior thermogenic fraction. This would be especially useful for distinguishing between thermogenic and nonthermogenic sources with similar spatial patterns (e.g., natural gas distribution and sewers).

## Data Availability

Aircraft measurement data^60^ used in this study are available from the Harvard Dataverse at 10.7910/DVN/UECQXX. The high-resolution inventory^61^ is available at https://doi.org/10.18434/mds2-2915.

## Supporting Information Available

Prior and posterior emission rates from individual ensemble members (XLSX)
More granular details on the inventory sector/subsector composition (including emission factors) and on the inventory construction, inventory versions, posterior emissions, and the results of an inversion parameter sensitivity test (PDF)
