# Supplementary material for: Underestimation of Thermogenic Methane Emissions in New York City
Source: Environ Sci Technol. 2024 May 14;58(21):9147–57. doi: 10.1021/acs.est.3c10307 (PMC11137862; doi:10.1021/acs.est.3c10307)
Supplement: Supplementary file 2 — es3c10307_si_002.pdf [file es3c10307_si_002.pdf]

Supporting information for "Underestimation of thermogenic methane emissions in New York City"

Joseph R. Pitt[1,§,], Israel Lopez-Coto[2,1,]*, Anna Karion[2], Kristian D. Hajny[1,3], Jay Tomlin[3], Robert Kaeser[3], Thilina Jayarathne[3,†], Brian H. Stirm[4], Cody R. Floerchinger[5], Christopher P. Loughner[6], Róisín Commane[7], Conor K. Gately[5,8,‡], Lucy R. Hutyra[8], Kevin R. Gurney[9], Geoffrey S. Roest[9], Jianming Liang[10], Sharon Gourdji[2,^], Kimberly L. Mueller[2], James R. Whetstone[2], Paul B. Shepson[1,3]*

[1]School of Marine and Atmospheric Sciences, Stony Brook University, Stony Brook, NY 11794, USA

[2]National Institute of Standards and Technology, Gaithersburg, MD 20899, USA

[3]Department of Chemistry, Purdue University, West Lafayette, IN 47907, USA

[4]School of Aviation and Transportation Technology, Purdue University, West Lafayette, IN 47906, USA

[5]Department of Earth and Planetary Sciences, Harvard University, Cambridge, MA 02138, USA

[6]Air Resources Laboratory, NOAA, College Park, MD 20740, USA

[7]Dept. of Earth and Environmental Sciences, Lamont-Doherty Earth Observatory, Columbia University, Palisades, NY 10964, USA

[8]Department of Earth and Environment, Boston University, Boston, MA 02215, USA

[9]School of Informatics, Computing and Cyber Systems, Northern Arizona University, Flagstaff, AZ 86011, USA

[10]Environmental Systems Research Institute, Redlands, CA 92373, USA

[§]Current address: School of Chemistry, University of Bristol, Bristol, BS8 1TS, UK

[†]Current address: Bristol Myers Squibb, New Brunswick, NJ 08901, USA

[‡]Current address: Metropolitan Area Planning Council, Boston, MA 02111, USA

[^]Current address: Moody's RMS, Detroit, MI 48214, USA

Email: joseph.pitt@bristol.ac.uk, israel.lopezcoto@nist.gov

Number of pages: 30 Number of figures: 9 Number of tables: 6

S1: Inventory methodological details

This section contains five tables that provide extra information regarding the high-resolution inventory (d03 domain). Details of each table are given in the corresponding caption. Below we include descriptions of:

- emissions from natural gas transmission (not associated with compressor stations).
- the inland water fluxes (rivers and lakes).
- the calculation of natural fluxes (wetlands and inland waters) within the d01 domain.
- a list of the “other” sectors taken straight from the gridded Environmental Protection Agency (GEPA) inventory.[1]
- the methodology used to estimate the number of people using onsite wastewater treatment systems within each state.

Natural gas transmission

Emissions from natural gas transmission not associated with compressor stations include the following sub-sectors from the EPA NIR[2]: Pipeline Leaks; M&R (Trans. Co. Interconnect); M&R (Farm Taps + Direct Sales); and Pipeline Venting. Emissions from these sub-sectors were allocated uniformly along transmission pipelines, using pipeline locations published by the EIA.[3] The emission rate per unit length of pipeline (given in Table S1.2) was calculated by dividing the total national emissions for these four sub-sectors by the total length of pipeline reported in the EPA NIR.[2]

Inland waters

Locations of rivers and lakes were taken from the National Wetlands Inventory (NWI).[4] Rosentreter et al.[5] reported median lake fluxes that depend strongly on lake size, with much larger fluxes from smaller lakes. However, McDonald et al.[6] showed that large lakes (> 1 km^2) constitute 71 % of the total lake area in the contiguous US, rising to 90 % if the Great Lakes are included. Therefore, all lake classes in the NWI (i.e., classes beginning with L) were assigned a flux of 5.00 $gCH_4\ m^{-2}\ yr^{-1}$, given as the median flux for lakes larger than 1 km^2 by Rosentreter et al.[5]. Similarly, all river classes in the NWI (i.e., classes beginning with R) were assigned a flux of 7.88 $gCH_4\ m^{-2}\ yr^{-1}$, given as the median flux for rivers by Rosentreter et al.[5]

Natural emissions in the d01 domain

In the large, coarse, d01 domain, anthropogenic emissions were taken from the GEPA (regridded to 0.08° × 0.08° using a conservative regridding scheme, described by Pitt et al.).[7,8] Natural emissions (i.e., wetlands and inland waters) in the d01 domain were calculated (on a 0.08° × 0.08° grid) following the same approach as used for the d03 natural emission maps. Emissions from rivers and lakes were only calculated for the US part of the domain (as the NWI is not available for Canada). Canadian wetland emissions were calculated based on cold-season values from WetCHARTs v1.3.1,[9] spatially downscaled using the 2015 Land Cover of Canada.[10] In the US, the wetland emission map used for the d01 domain corresponded to the wetland map used for the d03 domain for a given model simulation. However, Canadian emissions were based on this WetCHARTs-derived emission map in all cases (i.e., even when US wetland emissions were derived based on SOCCR1[11]/SOCCR2[12,13] fluxes and NWI[4] land cover), but with Canadian emissions rescaled by the ratio of the mean flux within the US part of the d01 domain to the mean WetCHARTS-derived flux for the same area. This ensured that there were no spurious step changes in wetland emission magnitude at the US-Canada border when US emissions were calculated based on SOCCR1 or SOCCR2 values.

Other emissions

The "Other" sector (see Table S1.1) consists of a number of minor-emitting sub-sectors that were taken directly from the GEPA.[1] These sectors were labelled in the GEPA as:

- 1A_Combustion_Mobile
- 1B1a_Abandoned_Coal
- 1B1a_Coal_Mining_Surface
- 1B1a_Coal_Mining_Underground
- 1B2a_Petroleum
- 1B2b_Natural_Gas_Processing
- 1B2b_Natural_Gas_Production
- 2B5_Petrochemical_Production
- 2C2_Ferroalloy_Production
- 4A_Enteric_Fermentation
- 4B_Manure_Management

- 4C_Rice_Cultivation
- 4F_Field_Burning
- 5_Forest_Firest
- 6D_Composting

Onsite wastewater treatment

To estimate the number of people using onsite systems at the state level, US Census state population estimates for 2019[14] were multiplied by an estimate of the fraction of people served by onsite systems. For New York state, this septic fraction estimate (16.1 %) was taken from the 2019 American Housing Survey.[15] Such recent data was not available for the other four states (CT, NJ, PA, DE) that intersect the domain. In those cases, the septic fraction reported in the 1990 US Census[16] (the last to provide this data at the individual state level) was used. To correct for recent changes in septic fraction, these state-level values from 1990 were multiplied by the ratio of whole-US septic fraction in 2019 (16.3 %; from the American Housing Survey) to whole-US septic fraction in 1990 (24.1 %).

Sector	No. of subsectors	Classification	No. of variants
Landfills	2	Non-thermogenic	1
Wetlands and inland waters	3	Non-thermogenic	3
Natural gas distribution	5	Thermogenic	6
Natural gas residential post meter	1	Thermogenic	6
Natural gas transmission	2	Thermogenic	1
Stationary combustion	4	Mixed	4
Wastewater	2	Non-thermogenic	2
Other (taken straight from GEPA)	15	Mixed	1

Table S1.1: Summary of the top-level sectoral breakdown of the high-resolution inventory, including the number of individual subsector maps that comprise each sector and the number of alternative variants constructed.

Sector	Sub-sector	Type	Emission factor	Reference
Natural gas distribution	M&R stations	M&R, inlet > 300 psig	2143 kg station^{-1}	EPA 2021[2]
		M&R, inlet 100-300 psig	995 kg station^{-1}	
		M&R, inlet < 100 psig	727 kg station^{-1}	
		Regulating, inlet > 300 psig	869 kg station^{-1}	
		Regulating, inlet 100-300 psig	143 kg station^{-1}	
		Regulating, inlet 40-100 psig	164 kg station^{-1}	
		Regulating, below grade	51 kg station^{-1}	
	Maintenance/upsets	Pressure relief valves	1.0 kg (mile of mains pipeline)$^{-1}$	EPA 2021[2]
		Pipeline blowdown	2.0 kg (mile of all pipeline)$^{-1}$	
		Mishaps	30.6 kg (mile of all pipeline)$^{-1}$	
	Service Pipelines	Unprotected steel	14.5 kg service^{-1}	EPA 2021[2]
		Cathodically protected steel	1.3 kg service^{-1}	
		Plastic	0.3 kg service^{-1}	
		Copper	4.9 kg service^{-1}	
	Consumer Meters	Residential	1.5 kg meter^{-1}	EPA 2021[2]
		Commercial	23.4 kg meter^{-1}	
		Industrial	105 kg meter^{-1}	
	Mains Pipelines	Bare steel	0.51 leaks mile^{-1}, 2.24 g min^{-1} leak^{-1}	Weller et al.[17]
		Cast iron	1.00 leaks mile^{-1}, 1.72 g min^{-1} leak^{-1}	
		Coated steel	0.61 leaks mile^{-1}, 2.00 g min^{-1} leak^{-1}	
		Plastic	0.43 leaks mile^{-1}, 2.03 g min^{-1} leak^{-1}	
Natural gas residential post meter			0.5 % of residential consumption	Fischer et al.[18]
Natural gas transmission	Compressor stations		1.09 mol/s/station (default)	EPA 2021[2]
	Other		1.20 µmol/m/s	

Table S1.2: Emission factors for natural gas sectors

Stationary combustion sub-sector	Fuel	EPA NIR correction	Gross calorific value to net calorific value	Energy unit (TJ MMBtu^{-1})	Emission factor (kg TJ^{-1})	Emission factor (g MMBtu^{-1})
Residential	Coal	--	--	--	--	--
	Petroleum	0.87	0.95	1/947.8	10	--
	Natural gas	--	--	--	--	--
	Wood	0.97	0.9	1/947.8	300	--
Commercial	Coal	1.02	0.95	1/947.8	10	--
	Petroleum	0.89	0.95	1/947.8	10	--
	Natural gas	1.0	0.9	1/947.8	5	--
	Wood	0.68	0.9	1/947.8	300	--
Industrial	Coal	0.46	0.95	1/947.8	10	--
	Petroleum	0.24	0.95	1/947.8	3	--
	Natural gas	0.89	0.9	1/947.8	1	--
	Wood	0.90	0.9	1/947.8	30	--
Electricity Production	Coal	1.04	0.95	1/947.8	1	--
	Petroleum	0.22	0.95	1/947.8	3	--
	Natural gas	0.99	--	--	--	5.4
	Wood	0.15	0.9	1/947.8	30	--

Table S1.3: Multiplicative factors used to convert from SEDS energy consumption estimates to annual CH_4 emissions for the stationary combustion subsectors. Emission factors in kg TJ^{-1} are default IPCC[19] values, while the emission factor for electricity production from natural gas (g MMBtu^{-1}) is taken from Hajny et al.[20] There is no reported residential coal use, and emissions from residential natural gas use are considered in the separate sector: Natural gas residential post meter.

Sector	Sub-sector	Type	Emission factor	Reference
Wetlands and inland waters	Wetland	See Table S1.5	See Table S1.5	See Table S1.5
	River		21.6 mg m^{-2} day^{-1}	Rosentreter et al.[5]
	Lake		13.7 mg m^{-2} day^{-1}	
Landfills	Municipal	Reporting to GHGRP	N/A (GHGRP emissions)	GHGRP[21]
		Not reporting to GHGRP	0.52 mol s^{-1} $landfill^{-1}$	EPA 2021[2]; LMOP[22]
	Industrial		N/A (GEPA emissions)	GEPA[1]
Wastewater	Domestic	Centralised (treatment plants)	0.019 mol s^{-1} (million gallons day^{-1})$^{-1}$	EPA 2021[2]; CWNS 2012[23]
		Onsite (e.g., septic tanks)	10.7 g day^{-1} (person using onsite system)$^{-1}$	EPA 2021[2]
	Industrial		N/A (GHGRP emissions)	GHGRP[21]

Table S1.4: Emission factors for non-thermogenic sectors.

Wetland Type	NWI code starting	SOCCR1 flux ($g\ m^{-2}\ yr^{-1}$)	SOCCR2 flux ($g\ m^{-2}\ yr^{-1}$)
Intertidal	E2 or M2	1.3	20.44
Terrestrial forested	PFO	7.6	24.76
Terrestrial non-forested	P (except PFO)	7.6	26.34

Table S1.5: Fluxes for the different wetland types calculated using data from the First State of the Carbon Cycle Report (SOCCR1)[11] and the Second State of the Carbon Cycle Report (SOCCR2).[12,13]

S2: Comparison of high-resolution inventory versions

A comparison between selected versions of the high-resolution inventory and a selection of lower resolution (0.1°) pre-existing inventories is shown in Figure S2.1. Here we have focussed on the correlation coefficient (r^2) as a measure of the accuracy of the spatial distribution of emissions. An accurate prior spatial distribution is important in any inversion, but it is especially so for the sectoral inverse modelling approach, in which the spatial distribution for the three components (urban area thermogenic emissions, urban area non-thermogenic emissions, and emissions outside the urban area) are fixed.

The four versions of the high-resolution inventory that have been used in the analysis presented in the main manuscript are indicated in Figure S2.1 as "HRA", "HRB", "HRC" and "HRD". A standardised naming convention is followed, whereby high-resolution inventory versions are denoted *AA_BBB_CC_DD*:

- *AA* denotes the spatial proxy used for natural gas distribution/post-meter emissions
    - A1 = state-level emissions distributed using ACES
    - A5 = 5-state-level emissions distributed using ACES
    - AL = LDC-level emissions distributed using ACES
    - V1 = state-level emissions distributed using Vulcan
    - V5 = 5-state-level emissions distributed using Vulcan
    - VL = LDC-level emissions distributed using Vulcan
- *BBB* denotes the spatial proxy used for stationary combustions emissions:
    - AS1 = state-level emissions distributed using ACES
    - AS5 = 5-state-level emissions distributed using ACES
    - VS1 = state-level emissions distributed using Vulcan
    - VS5 = 5-state-level emissions distributed using Vulcan
- *CC* denotes the wetland emission map:
    - WC = WetCHARTs fluxes downscaled using NLCD landcover
    - S1 = SOCCR1 fluxes applied to NWI wetland classes
    - S2 = SOCCR2 fluxes applied to NWI wetland classes
- *DD* denotes the onsite wastewater treatment emission level:
    - SN = National emissions distributed using NLCD landcover
    - SS = State-level emissions distributed using NLCD landcover

Anthropogenic emissions from the pre-existing inventories were combined with natural emissions (wetlands, rivers and lakes) from the high-resolution inventory prior to this analysis. The wetland emission maps used in each case are denoted following the same convention as used for the high-resolution inventory versions (see code description above). The anthropogenic emissions are denoted as follows:

- ED4 = anthropogenic emissions from EDGAR v4.2[24]
- ED5 = anthropogenic emissions from EDGAR v5[25,26]
- EPA = anthropogenic emissions from the GEPA[1]

Fluxes within the d01 domain were also included in the calculation of the modelled timeseries, for all cases shown in Figure S2.1 (i.e., both the high-resolution and pre-existing inventories). Anthropogenic emissions in d01 were taken from the GEPA in all cases; see SI Section S1 for a description of how d01 natural emissions were calculated.

Each boxplot consists of results from the 9 flights (after averaging across the transport model ensemble for each flight). The vast majority of high-resolution inventory versions resulted in higher mean and median correlation coefficients than the pre-existing inventories. The analysis in the main paper focusses on the high-resolution inventory versions HRA, HRB, HRC and HRD. HRA and HRC were selected on the basis that they had the highest mean and median r^2 values, respectively. In principle it should be more accurate to calculate natural gas, stationary combustion and onsite wastewater treatment emissions over the smallest possible spatial area before disaggregating using a spatial proxy. After filtering according to these criteria, we also wanted to carry forward one prior where Vulcan had been used as a spatial proxy and another where ACES had been used as a spatial proxy. HRB and HRD were therefore selected because they had the highest median r^2 values of the filtered versions that used ACES and Vulcan respectively.

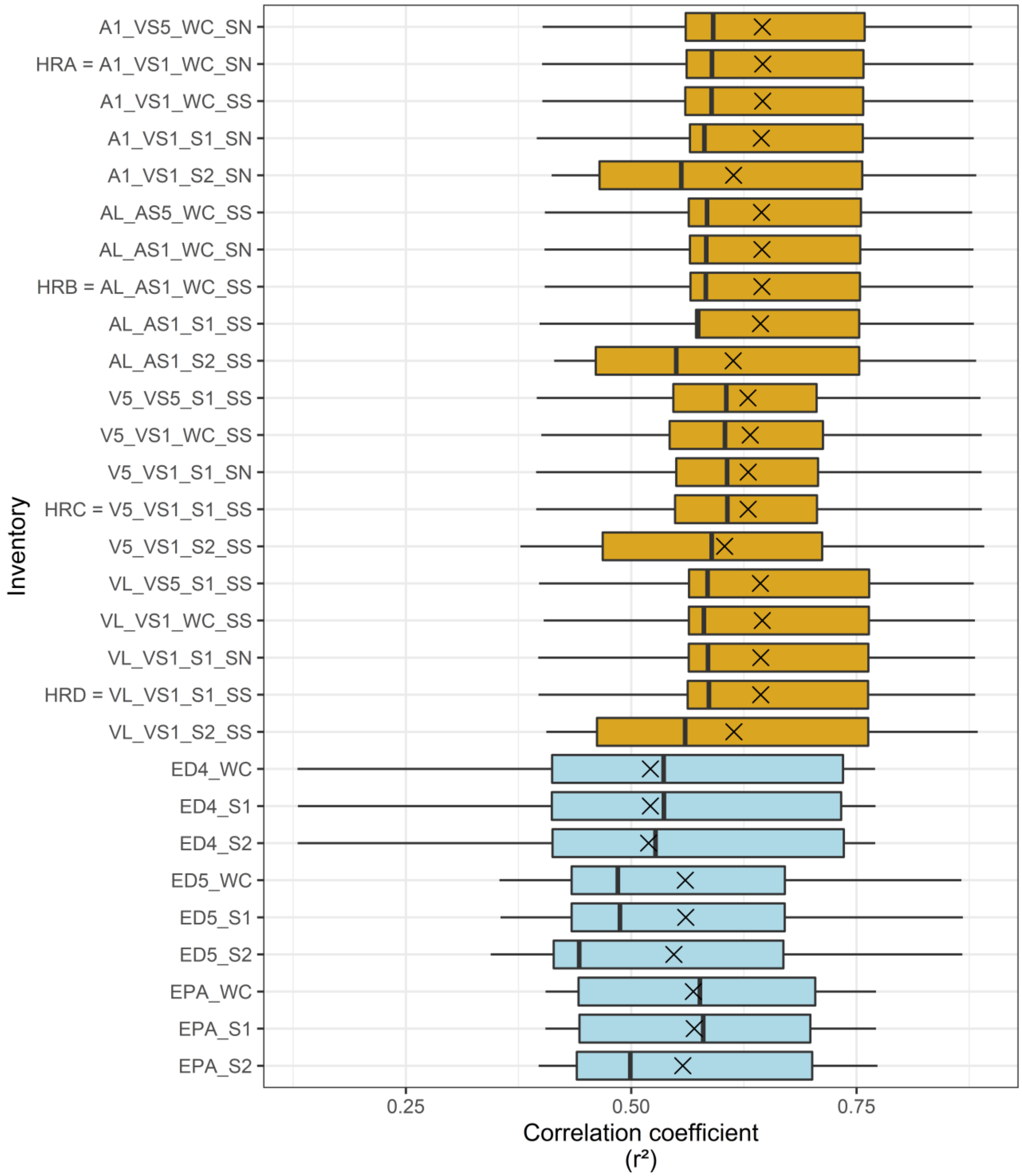

Figure S2.1: Correlation coefficient between the measured mole fraction timeseries and modelled mole fraction timeseries using a variety of d03 inventory (prior) emissions. This plot contains a subset of the 144 possible combinations of the high-resolution inventory (gold bars) as well as a selection of lower resolution (0.1°) pre-existing inventories (blue bars). Boxplot convention is described in the caption of Figure S5.4.

S3: Aircraft measurements

The CH_4 mole fraction measurements used in this study were made using a Picarro* Cavity Ringdown Spectrometer (either model G2301-f or G2301-m, depending on the flight).[27] These measurements are traceable to the WMO X2004A CH_4 scale[28] via the in-flight sampling of three calibration cylinders, provided by NOAA, with a typical precision of 3 nmol mol^{-1} for CH_4. The data acquisition interval was between 1.2 and 2.3 seconds, depending on the specific analyser used on a given flight. The flights were all conducted during the months of November, February or March. The flight tracks, flight dates and flight times are shown in Figure S3.1, along with the aggregate footprint for each flight.

*Certain commercial equipment, instruments, or materials are identified in this paper in order to specify the experimental procedure adequately. Such identification is not intended to imply recommendation or endorsement by NIST nor is it intended to imply that the materials or equipment identified are necessarily the best available for the purpose.

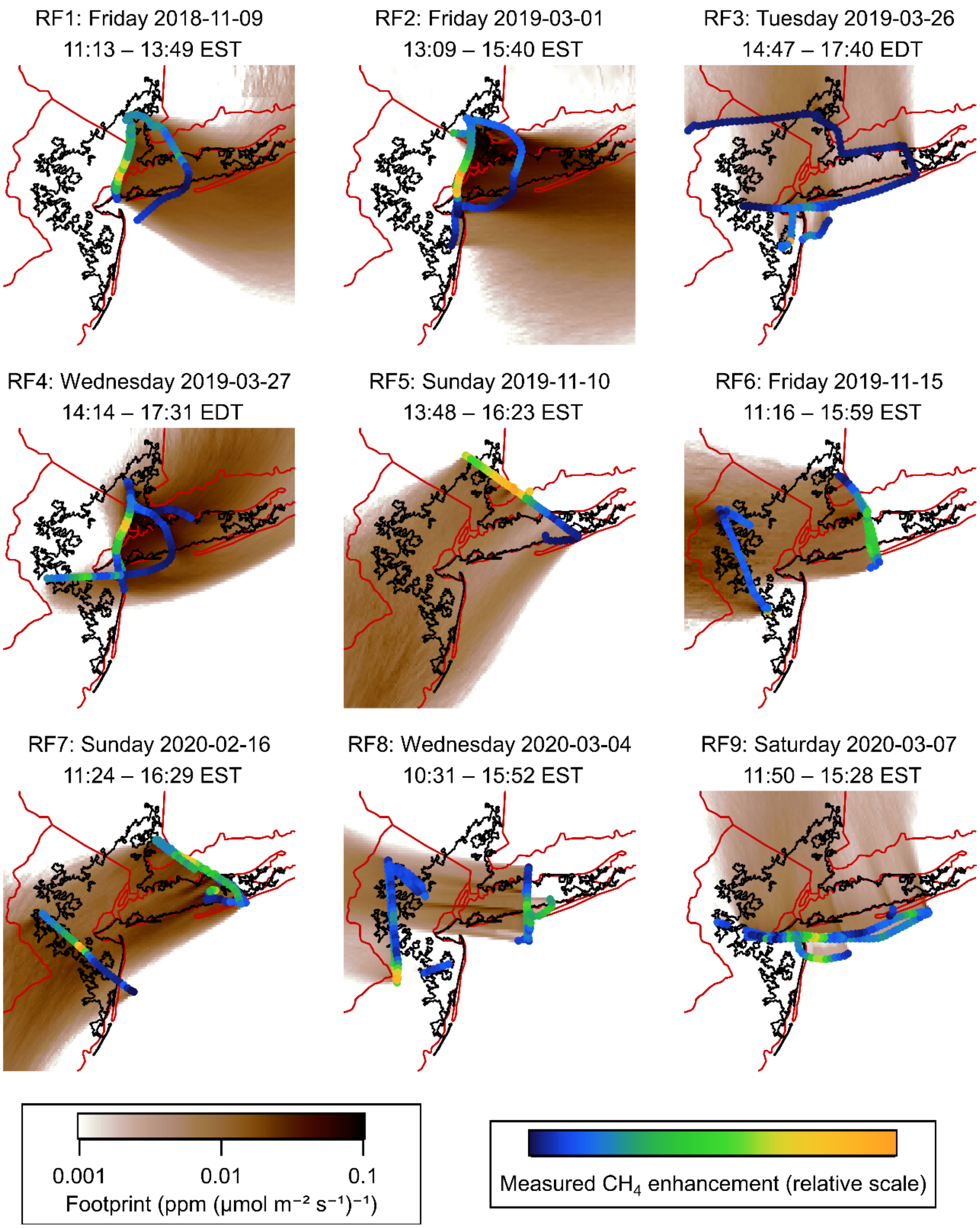

Figure S3.1: Flight tracks for each day, coloured by measured CH_4 enhancement. The aggregate footprint for each flight is also shown (using ERA5 meteorology and Kantha and Clayson[29] turbulence parameterisation), using a logarithmic scale saturated at the limits indicated. State boundaries are shown in red and the NY-UA is outlined in black. Flight times are given in local time. This figure has been adapted from Pitt et al.,[7] reprinted with permission from University of California Press, Copyright © 2022 (https://creativecommons.org/licenses/by/4.0/).

S4: Inverse modelling

The outer d01 and nested d03 domains are shown in Figure S4.1. The higher resolution within the d03 domain can clearly be seen. The timeseries of modelled mole fractions ($\boldsymbol{\lambda}\mathbf{Y}_{\boldsymbol{mod}}$) only includes contributions from within the model domains (i.e., both d01 and d03). It is therefore necessary to estimate the measured mole fraction enhancements that are solely attributable to emissions within these domains ($\boldsymbol{y}_{\boldsymbol{enh}}$), so that the difference between the two can be used in the cost function (equation 1 in the main manuscript). This is achieved by subtracting a background term from the measured mole fraction timeseries ($\boldsymbol{y}_{\boldsymbol{tot}}$), representing all other influences on this measured timeseries, according to the following equation:

$$\boldsymbol{y}_{\boldsymbol{enh}} = \boldsymbol{y}_{\boldsymbol{tot}} - \left(\overline{\boldsymbol{y}}_{\boldsymbol{bg}} - \overline{(\boldsymbol{\lambda}_{\boldsymbol{b}}\mathbf{Y}_{\boldsymbol{mod}})}_{\boldsymbol{bg}}\right) \quad \text{(S1)}$$

Here $\overline{\boldsymbol{y}}_{\boldsymbol{bg}}$ is the average measured mole fraction taken over a set of points defined as "background points", and $\overline{(\boldsymbol{\lambda}_{\boldsymbol{b}}\mathbf{Y}_{\boldsymbol{mod}})}_{\boldsymbol{bg}}$ is the average modelled mole fraction taken over a set of background points. The term $\overline{(\boldsymbol{\lambda}_{\boldsymbol{b}}\mathbf{Y}_{\boldsymbol{mod}})}_{\boldsymbol{bg}}$ is required to account for the influence of sources within the model domains on $\overline{\boldsymbol{y}}_{\boldsymbol{bg}}$. These background points are selected so as to minimise the influence of emission sources within the domains on the measured and modelled mole fractions at these points, while avoiding outlier points (e.g., those impacted by entrainment of free-tropospheric air during a given minute of the flight). Therefore, the background points for $\overline{\boldsymbol{y}}_{\boldsymbol{bg}}$ are defined as all points whose mole fractions lie between the 1st and 5th percentiles of the measured timeseries. Similarly the background points for $\overline{(\boldsymbol{\lambda}_{\boldsymbol{b}}\mathbf{Y}_{\boldsymbol{mod}})}_{\boldsymbol{bg}}$ are defined as all points whose mole fractions lie between the 1st and 5th percentiles of the modelled timeseries. This definition corresponds to one of the sensitivity tests conducted by Pitt et al.;[7] in this study it was found to yield very similar posterior results to the base case (under which a single set of background points was defined). See Pitt et al.[7] for further discussion of the different possible background choices and the corresponding sensitivity test results.

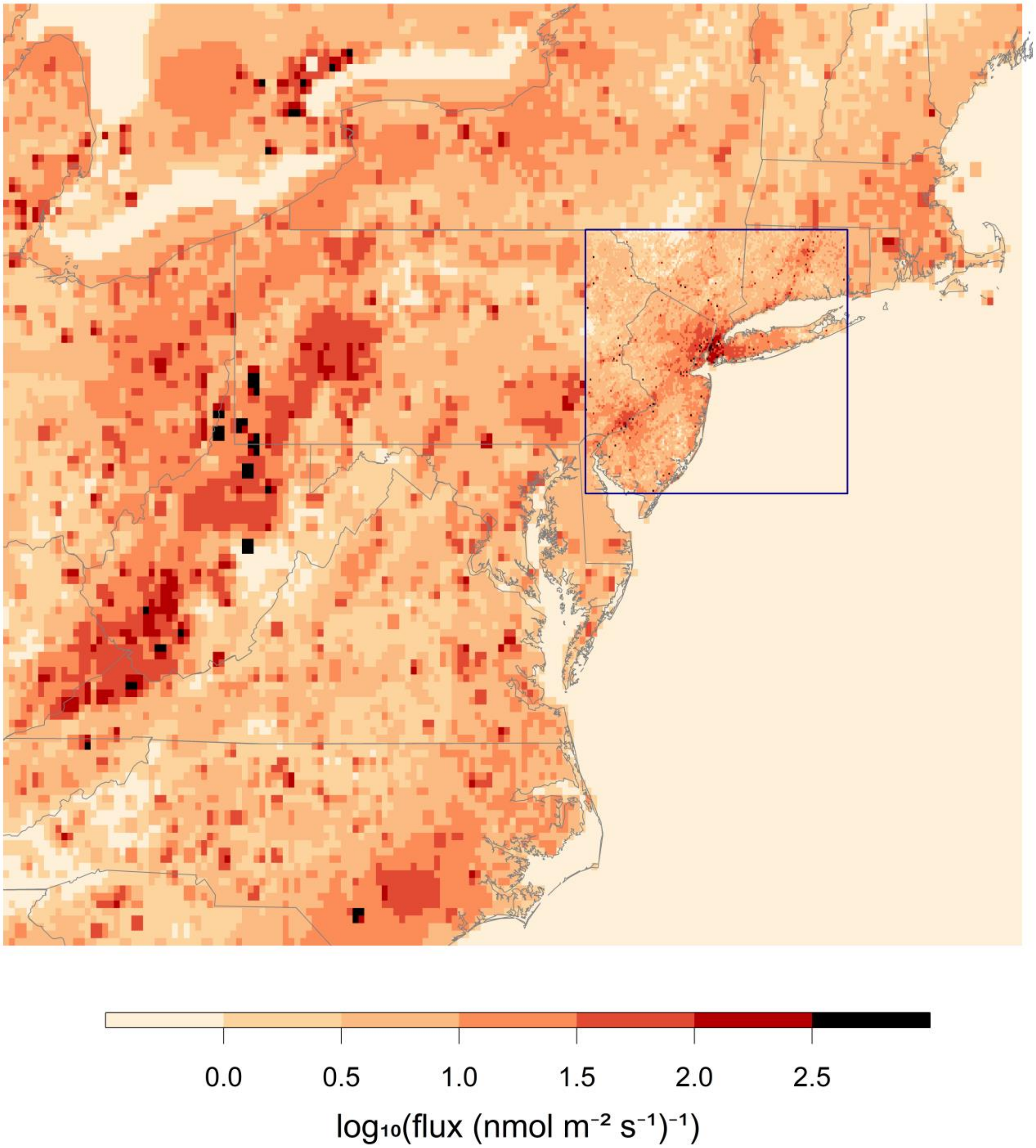

Figure S4.1: A map showing fluxes within the d01 domain (entire plot) and the d03 domain (blue box). The high-resolution inventory version shown here is version HRB.

S5: Results and sensitivity tests

Emission maps for individual sectors are shown in Figures S5.1 and S5.2. Note that a log scale is used so that the spatial patterns of smaller sources can also be seen.

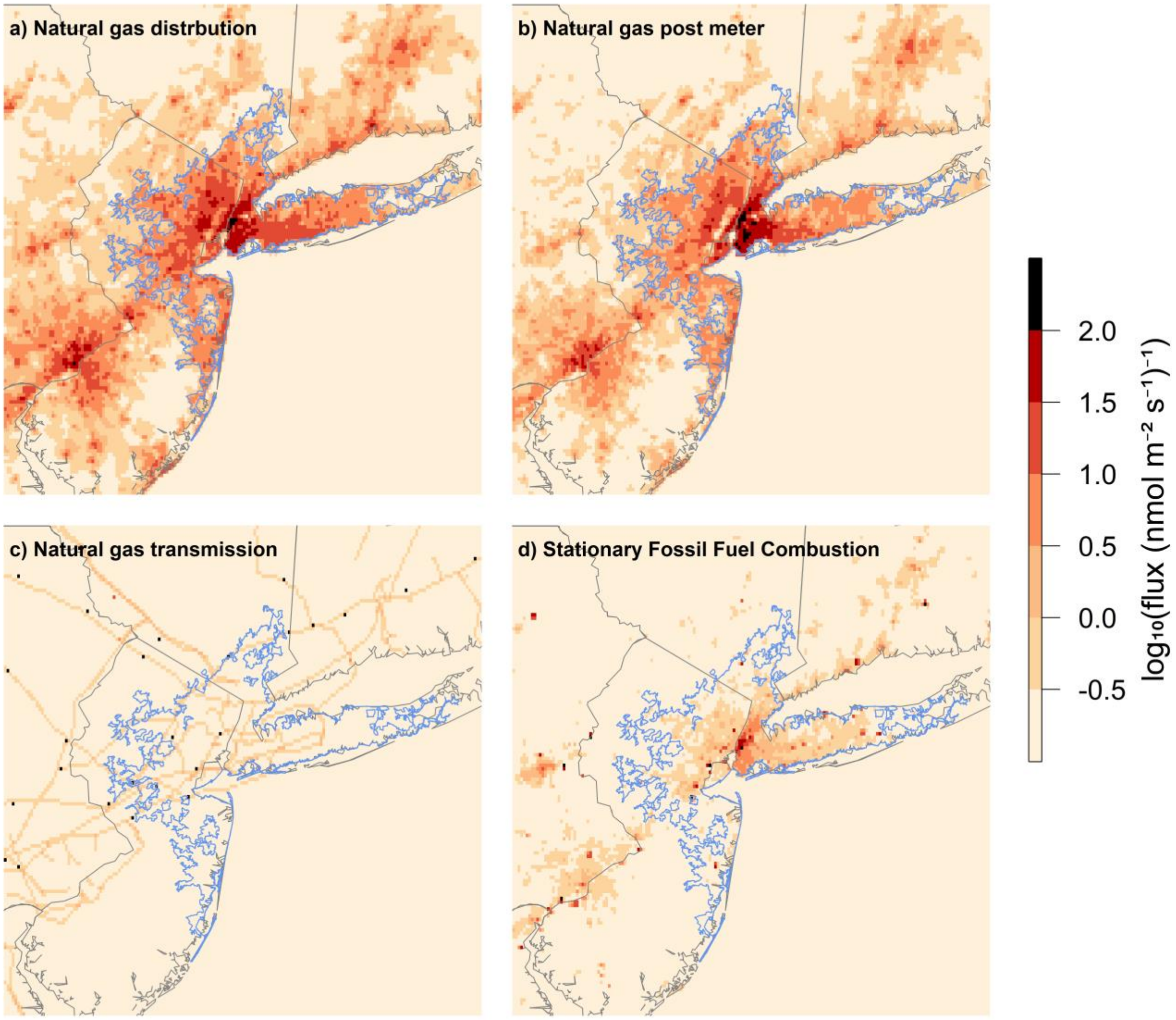

Figure S5.1: Panel plot showing flux maps for the individual thermogenic sectors. All plots show fluxes on a logarithmic scale. The NY-UA outline is shown in blue. The high-resolution inventory version shown here is version HRB.

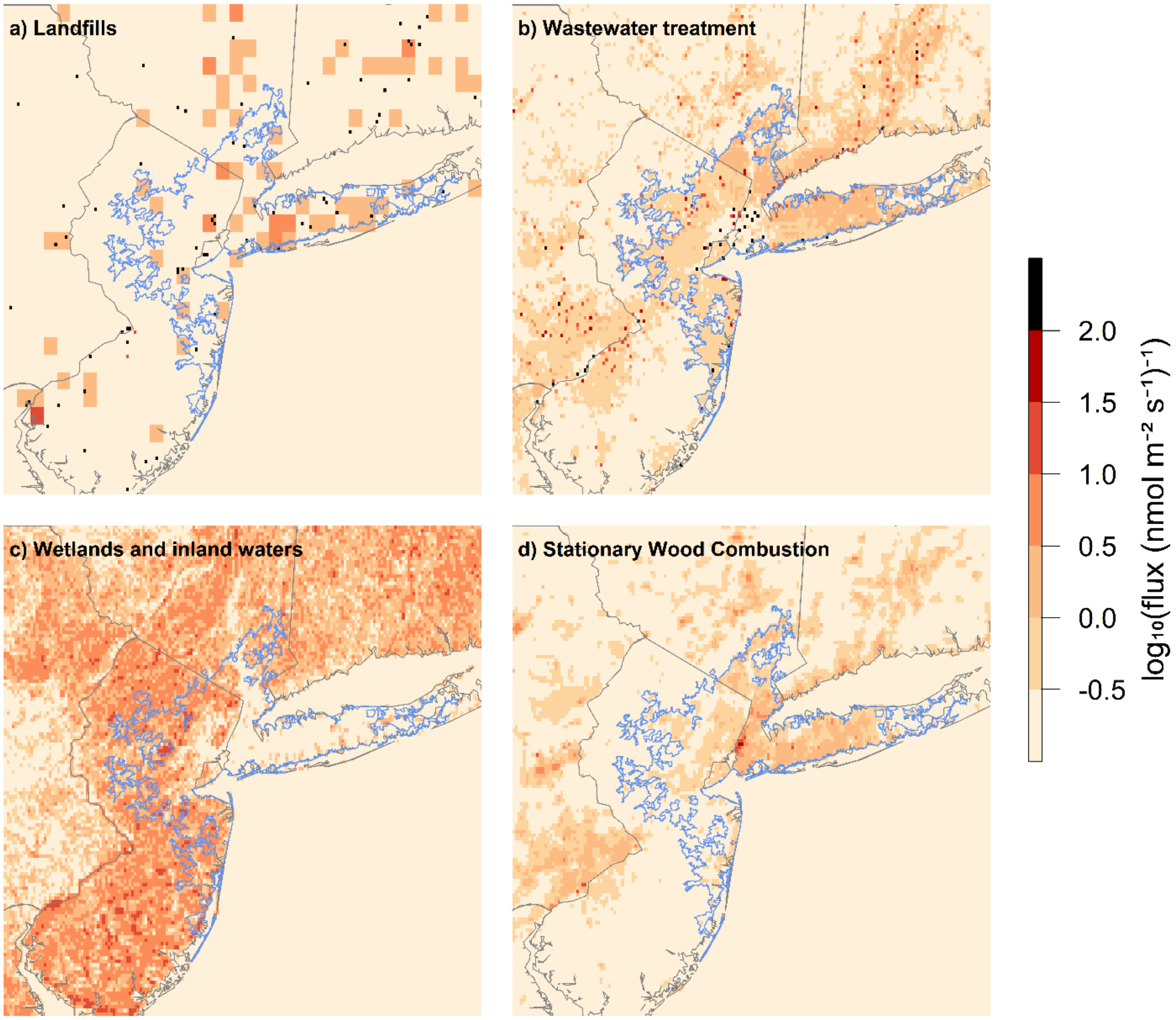

Figure S5.2: Panel plot showing flux maps for the individual non-thermogenic sectors. All plots show fluxes on a logarithmic scale. The NY-UA outline is shown in blue. The high-resolution inventory version shown here is version HRB.

A comparison of emission rates for the New York-Newark urban area (NY-UA) based on the GEPA, the high-resolution inventory (four versions) and the posterior sectoral and spatial inversion results is shown in Figure S5.3. The thermogenic fraction is also shown in all cases where available. The inventory totals by sector are also given in Table S5.1.

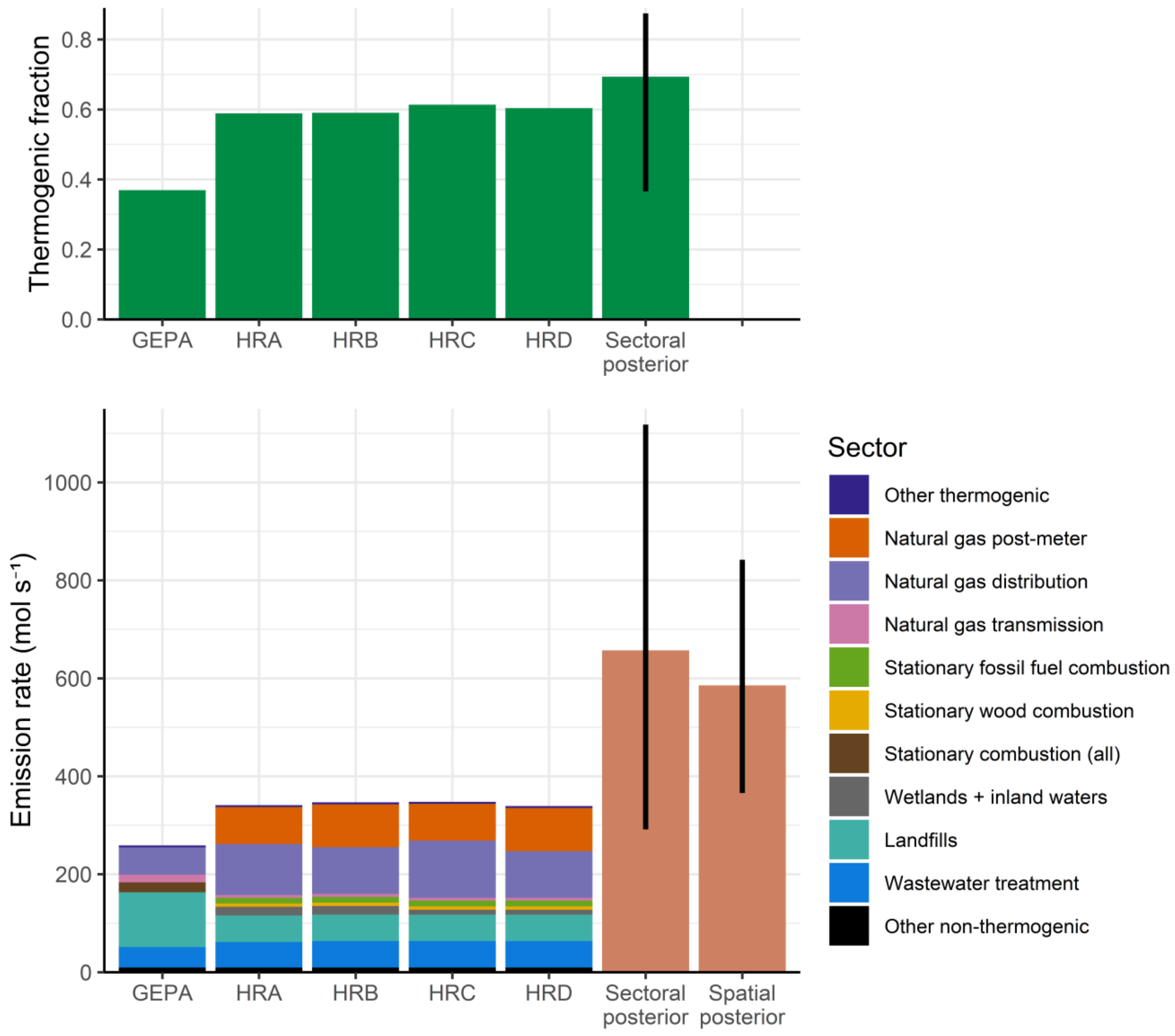

Figure S5.3: Thermogenic emission fraction (upper panel) and total emission rate (lower panel) for the NY-UA. Values are shown for the GEPA, four versions of the high-resolution inventory and the posterior results of both inversions. Error bars represent flight-to-flight variability for the posterior results, calculated as 95 % confidence intervals.

<table>
<tr><th></th><th>GEPA</th><th>HRA</th><th>HRB</th><th>HRC</th><th>HRD</th></tr>
<tr><td>Natural gas post-meter</td><td>N/A</td><td>75.50</td><td>87.49</td><td>75.22</td><td>87.93</td></tr>
<tr><td>Natural gas distribution</td><td>55.58</td><td>103.72</td><td>95.41</td><td>116.61</td><td>95.25</td></tr>
<tr><td>Natural gas transmission</td><td>16.05</td><td>5.95</td><td>5.95</td><td>5.95</td><td>5.95</td></tr>
<tr><td>Stationary fossil fuel combustion</td><td rowspan="2">19.93</td><td>11.47</td><td>11.68</td><td>11.47</td><td>11.47</td></tr>
<tr><td>Stationary wood combustion</td><td>6.94</td><td>6.93</td><td>6.94</td><td>6.94</td></tr>
<tr><td>Wetlands + inland waters</td><td>N/A</td><td>17.45</td><td>17.45</td><td>9.86</td><td>9.86</td></tr>
<tr><td>Landfills</td><td>112.00</td><td>54.26</td><td>54.26</td><td>54.26</td><td>54.26</td></tr>
<tr><td>Wastewater treatment</td><td>41.77</td><td>52.18</td><td>53.92</td><td>53.92</td><td>53.92</td></tr>
<tr><td>Other thermogenic</td><td>4.10</td><td>4.10</td><td>4.10</td><td>4.10</td><td>4.10</td></tr>
<tr><td>Other non-thermogenic</td><td>9.49</td><td>9.49</td><td>9.49</td><td>9.49</td><td>9.49</td></tr>
<tr><td>Total</td><td>258.91</td><td>341.06</td><td>346.68</td><td>347.82</td><td>339.18</td></tr>
</table>

Table S5.1: Sectoral emission totals in mol s^{-1} for the NY-UA according to the GEPA and four versions of the high-resolution inventory. Note that the GEPA does not separate stationary combustion by fossil fuel and wood, so the combined total is reported here.

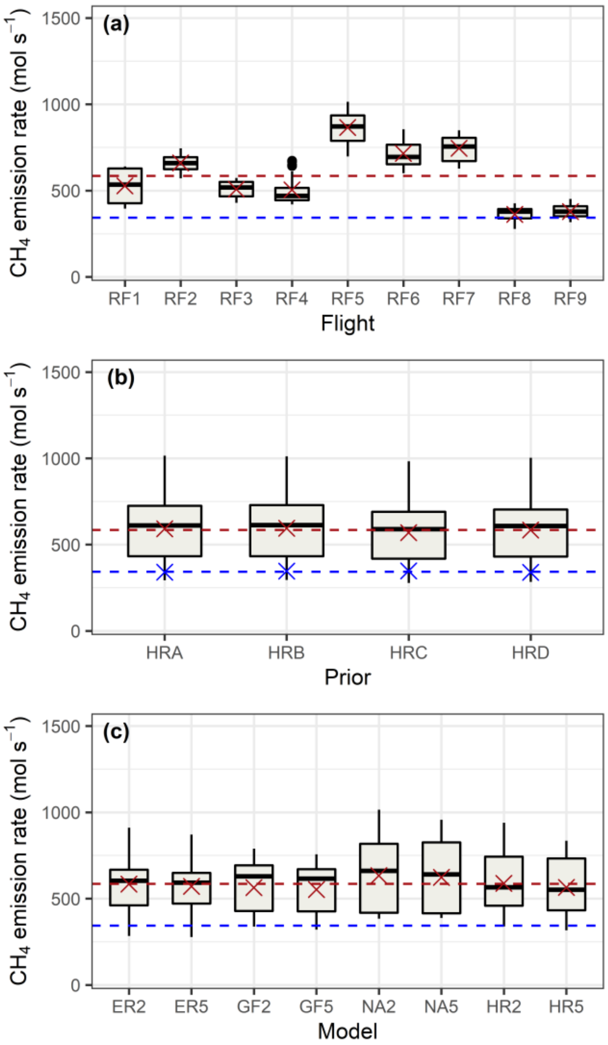

Figure S5.4: Posterior spatial inversion emission rates for the NY-UA, broken down by: (a) flight, (b) prior and (c) transport model. Mean posterior results for each boxplot are shown as red crosses, with the overall mean shown as a dashed red line. Mean prior values are shown in blue following the same convention. Transport models are labelled as follows: ER is ERA5, GF is GFS, HR is HRRR, and NA is NAM. The 2 and 5 represent the Kantha and Clayson[29] and Hanna[30] turbulence parameterisations, respectively. Boxplot convention follows Pitt et al.[7]: "The boxes extend between the upper and lower quartiles, with the median values shown as solid horizontal black bars. The whiskers extend to the highest and lowest data points within 1.5 times the interquartile range of the upper and lower quartiles, respectively. All data outside these whiskers are shown as individual points."

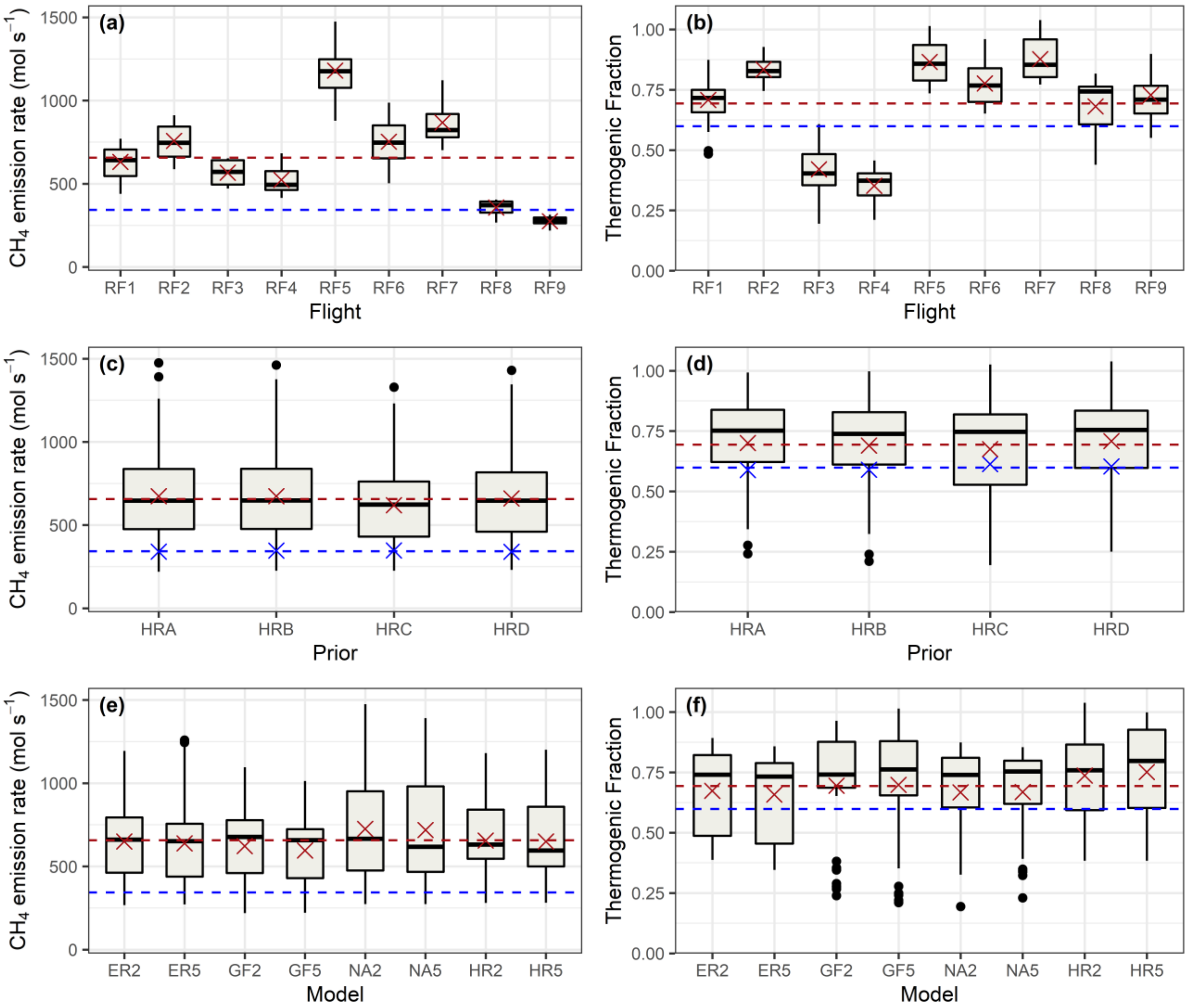

Figure S5.5: Posterior sectoral inversion results for the NY-UA. Panels (a, c, e) show total emission rates, while panels (b, d, f) show the fraction of emissions from thermogenic sources. Mean posterior results for each boxplot are shown as red crosses, with the overall mean shown as a dashed red line. Mean prior values are shown in blue following the same convention. Transport models are labelled as follows: ER is ERA5, GF is GFS, HR is HRRR, and NA is NAM. The 2 and 5 represent the Kantha and Clayson[29] and Hanna[30] turbulence parameterisations, respectively. Boxplot convention is described in the caption of Figure S5.4.

Statistical analysis of prior and posterior timeseries

We calculated several statistics (correlation coefficient, mean difference, standard deviation of the difference) to assess the agreement between the measured enhancements and the modelled timeseries based on the prior, sectoral posterior and spatial posterior emission maps. The measured enhancements correspond to $\boldsymbol{y_{enh}}$ from equation S1, and the modelled timeseries include contributions from both the d01 and d03 domains. When calculating the posterior modelled timeseries, one can either multiply the footprints for each transport model by the specific posterior emission map derived using that transport model, or one can use the transport-model-average posterior emission map for all footprints. Statistics calculated using both of these approaches are shown in Figure S5.6. In both cases the posterior timeseries calculated using the different transport model footprints are averaged for each flight, so that each individual box represents only the spread in a given statistic across the nine flights.

It is clear from these results that the posterior emission maps derived using both inversion approaches reduce the mean difference between the modelled timeseries and the measured enhancements relative to the prior. Overall they also reduce the standard deviation of this difference. The spatial posterior yields a higher correlation coefficient than the prior and the sectoral posterior. This can be expected, because the spatial inversion has greater freedom to spatially redistribute emissions relative to the sectoral inversion, so likely yields a more accurate posterior representation of the spatial distribution of emissions. Conversely, information regarding the relative magnitude of thermogenic and non-thermogenic emissions is lost in the spatial posterior, but retained in the sectoral posterior.

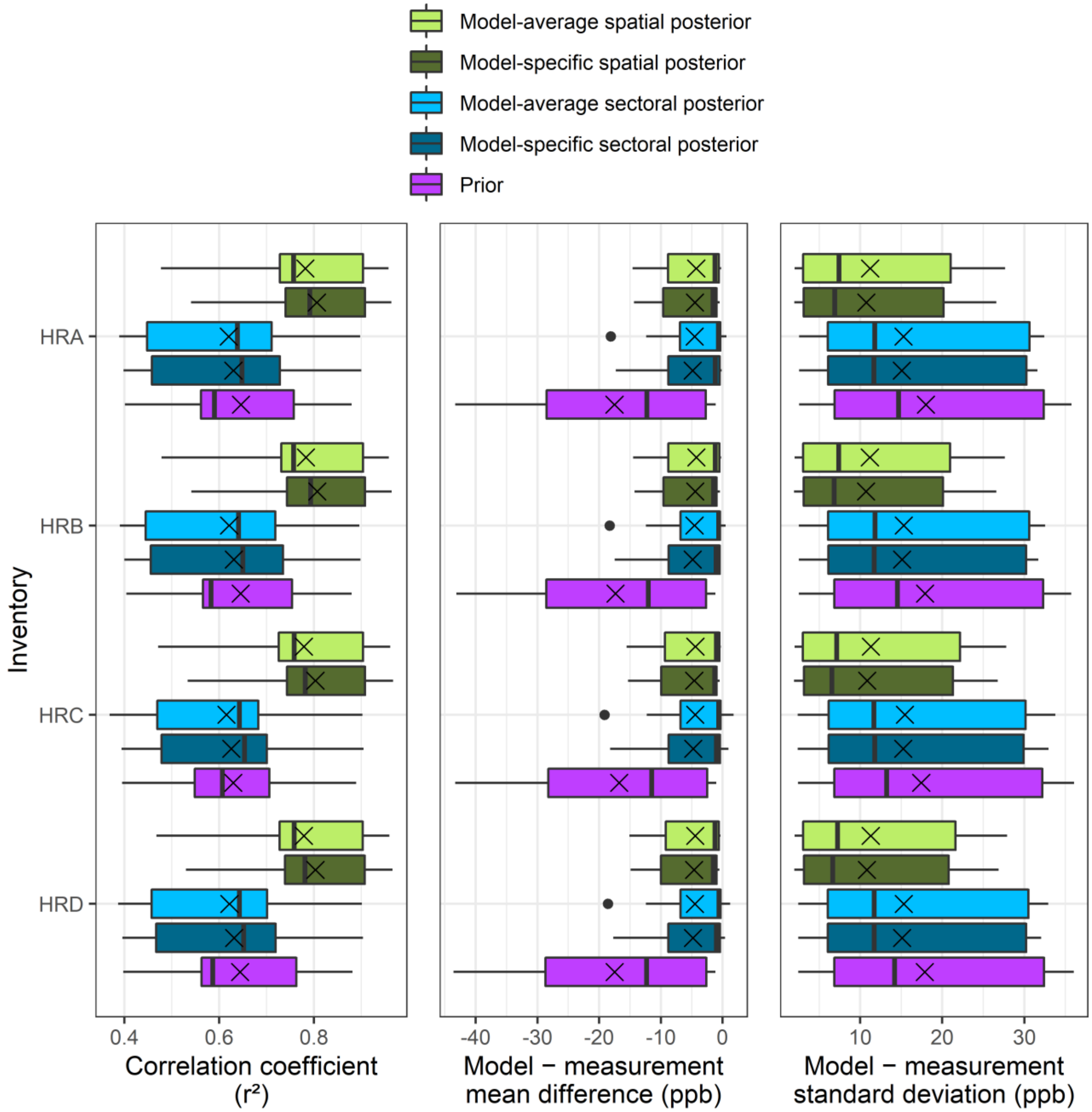

Figure S5.6: Statistics showing the difference between the measured enhancements and the modelled timeseries calculated using the prior, sectoral posterior and spatial posterior emission maps. The posterior emission maps are calculated by multiplying the footprint for each transport model by either the transport-model-specific posterior emission map, or the transport-model-average posterior emission map. The resulting posterior timeseries are then averaged across all transport models in both cases, such that each box represents only the spread in a given statistic across the nine flights.

Sensitivity test 1 – prior uncertainty

We conducted a sensitivity test to assess the impact of the prescribed prior uncertainty on the posterior results of the sectoral inversion. In the base case described in the main text, the prior uncertainty on the scaling factor for all three model components (urban area thermogenic, urban area non-thermogenic and outside contribution) was set to 0.5, representing a 1σ uncertainty of 50 % for each component. Our sensitivity test involved two alternative choices for this parameter: 0.25 and 1.0.

When the uncertainty on the prior scaling factor was set to 0.25, the posterior emission estimate was (610 ± 226) mol s^{-1}, and the posterior thermogenic fraction was 0.66 ± 0.09 (uncertainty quoted as 1σ flight-to-flight variability in all cases). Using a prior scaling factor uncertainty of 1.0 yielded posterior estimates for total emissions and thermogenic fraction of (670 ± 285) mol s^{-1} and 0.75 ± 0.30 respectively. Comparing these to our base case estimates of (657 ± 273) mol s^{-1} and 0.69 ± 0.19, it can be seen that larger, and more variable, estimates of posterior emissions and posterior thermogenic fraction were obtained when the prior constraints were relaxed (i.e., a larger prior uncertainty is used), as one would expect. However, the fact that the variability in the mean result induced by this choice is less than 8 % in all cases is an encouraging sign that the overall conclusions of this study are robust to reasonable changes in the specification of this parameter.

Sensitivity test 2 – CO_2 proxies

The publicly available version of ACES v2.0[31] was released during the writing of this manuscript – the analysis presented in this study used a pre-release version with some small differences. The Vulcan inventory used in this study is the annual version of Vulcan v3.0[32] – there is also an hourly version of Vulcan v3.0[33] available, whose annual totals are slightly different. We wanted to test if the main ensemble of priors used in our manuscript sufficiently represented the uncertainties in input data, so as to cover the small differences between these ACES and Vulcan versions. Thus, we created two additional inventory versions using the publicly available ACES v2.0 and the annual average of the hourly Vulcan v3.0. Both versions used LDC-level emissions for natural gas distribution (AL/VL), state-level emissions for stationary combustion (AS1/VS1) and national emissions for onsite wastewater treatment (SN). These specific combinations were chosen because they were the combinations that displayed the largest differences relative to the corresponding priors used in our original analysis (i.e. they represented the "worst case scenario"). The emission

rate for one compressor station was also updated in these new versions. These two anthropogenic versions were combined with the two different versions for wetlands and inland waters used in the main manuscript (S1 and WC), to create a total of four new versions for this sensitivity test.

We repeated our inverse modelling analysis using these four revised inventory versions, to test the impact on the posterior results and thus check if the uncertainty provided by the ensemble approach used is an appropriate representation of real uncertainties in bottom-up proxy data. We found that for the sectoral inversion, the results show a mere 1.27 % difference in total emissions and a 1.65 % difference in thermogenic fraction (referenced to the paper mean values), with both numbers well below the 1σ variabilities across priors of the original ensemble (3.9 % in total emissions and 2.1 % in thermogenic fraction). In addition, the correlation (r^2) among daily averages was 0.9999 for the total emissions and 0.9983 for the thermogenic fractions. Similarly, for the spatial inversion we found a 0.5 % difference in total emissions and a correlation (r^2) among daily averages of 0.9999. These results demonstrate that the prior ensemble approach, and the original ensemble members used, provided a good representation of the expected uncertainties due to activity data and spatial proxies (at least those coming from reasonable data updates as those seen in ACES and Vulcan).

References

(1) Maasakkers, J. D.; Jacob, D. J.; Sulprizio, M. P.; Turner, A. J.; Weitz, M.; Wirth, T.; Hight, C.; DeFigueiredo, M.; Desai, M.; Schmeltz, R.; Hockstad, L.; Bloom, A. A.; Bowman, K. W.; Jeong, S.; Fischer, M. L. Gridded National Inventory of U.S. Methane Emissions. *Environ. Sci. Technol.* **2016**, *50* (23), 13123–13133. https://doi.org/10.1021/acs.est.6b02878.

(2) EPA. *Inventory of U.S. Greenhouse Gas Emissions and Sinks: 1990-2019*; EPA, 2021.

(3) EIA. Natural Gas Interstate and Intrastate Pipelines https://www.eia.gov/maps/map_data/NaturalGas_InterIntrastate_Pipelines_US_EIA.zip (accessed May 28, 2021).

(4) US Fish & Wildlife Service. National Wetlands Inventory https://www.fws.gov/program/national-wetlands-inventory/data-download (accessed Jun 4, 2021).

(5) Rosentreter, J. A.; Borges, A. V.; Deemer, B. R.; Holgerson, M. A.; Liu, S.; Song, C.; Melack, J.; Raymond, P. A.; Duarte, C. M.; Allen, G. H.; Olefeldt, D.; Poulter, B.; Battin, T. I.; Eyre, B. D. Half of Global Methane Emissions Come from Highly Variable Aquatic Ecosystem Sources. *Nat. Geosci.* **2021**, *14* (4), 225–230. https://doi.org/10.1038/s41561-021-00715-2.

(6) McDonald, C. P.; Rover, J. A.; Stets, E. G.; Striegl, R. G. The Regional Abundance and Size Distribution of Lakes and Reservoirs in the United States and Implications for Estimates of Global Lake Extent. *Limnol. Oceanogr.* **2012**, *57* (2), 597–606. https://doi.org/10.4319/lo.2012.57.2.0597.

(7) Pitt, J. R.; Lopez-Coto, I.; Hajny, K. D.; Tomlin, J.; Kaeser, R.; Jayarathne, T.; Stirm, B. H.; Floerchinger, C. R.; Loughner, C. P.; Gately, C. K.; Hutyra, L. R.; Gurney, K. R.; Roest, G. S.; Liang, J.; Gourdji, S.; Karion, A.; Whetstone, J. R.; Shepson, P. B. New York City Greenhouse Gas Emissions Estimated with Inverse Modeling of Aircraft Measurements. *Elem. Sci. Anthr.* **2022**, *10* (1), 1–13. https://doi.org/10.1525/elementa.2021.00082.

(8) Zhuang, J. XESMF: Universal Regridder for Geospatial Data. 2020. https://doi.org/10.5281/zenodo.1134365.

(9) Bloom, A. A.; Bowman, K. W.; Lee, M.; Turner, A. J.; Schroeder, R.; Worden, J. R.; Weidner, R. J.; McDonald, K. C.; Jacob, D. J. CMS: Global 0.5-deg Wetland Methane

Emissions and Uncertainty (WetCHARTs v1.3.1). https://doi.org/10.3334/ORNLDAAC/1915.

(10) Government of Canada; Natural Resources Canada; Canada Centre for Remote Sensing. 2015 Land Cover of Canada https://open.canada.ca/data/en/dataset/4e615eae-b90c-420b-adee-2ca35896caf6 (accessed Jan 29, 2023).

(11) Bridgham, S. D.; Megonigal, J. P.; Keller, J. K.; Bliss, N. B.; Trettin, C. Wetlands - Supplemental Materials. In *The First State of the Carbon Cycle Report (SOCCR): The North American Carbon Budget and Implications for the Global Carbon Cycle*; King, A. W., Dilling, L., Zimmerman, G. P., Fairman, D. M., Houghton, R. A., Marland, G., Rose, A. Z., Wilbanks, T. J., Eds.; National Oceanic and Atmospheric Administration, National Climatic Data Center: Asheville, NC, USA, 2007; pp 177–192.

(12) Kolka, R.; Trettin, C.; Tang, W.; Krauss, K.; Bansal, S.; Drexler, J.; Wickland, K.; Chimner, R.; Hogan, D.; Pindilli, E. J.; Benscoter, B.; Tangen, B.; Kane, E.; Bridgham, S.; Richardson, C. Terrestrial Wetlands. In *Second State of the Carbon Cycle Report (SOCCR2): A Sustained Assessment Report*; Cavallaro, N., Shrestha, G., Birdsey, R., Mayes, M. A., Najjar, R. G., Reed, S. C., Romero-Lankao, P., Zhu, Z., Eds.; U.S. Global Change Research Program: Washington, DC, USA, 2018; pp 507–567. https://doi.org/10.7930/ SOCCR2.2018.Ch13.

(13) Windham-Myers, L.; Cai, W.-J.; Alin, S. R.; Andersson, A.; Crosswell, J.; Dunton, K. H.; Hernandez-Ayon, J. M.; Herrmann, M.; Hinson, A. L.; Hopkinson, C. S.; Howard, J.; Hu, X.; Knox, S. H.; Kroeger, K.; Lagomasino, D.; Megonigal, P.; Najjar, R. G.; Paulsen, M.-L.; Peteet, D.; Pidgeon, E.; Schäfer, K. V. R.; Tzortziou, M.; Wang, Z. A.; Watson, E. B. Tidal Wetlands and Estuaries. In *Second State of the Carbon Cycle Report (SOCCR2): A Sustained Assessment Report*; Cavallaro, N., Shrestha, G., Birdsey, R., Mayes, M. A., Najjar, R. G., Reed, S. C., Romero-Lankao, P., Zhu, Z., Eds.; U.S. Global Change Research Program: Washington, DC, USA, 2018; pp 596–648. https://doi.org/10.7930/SOCCR2.2018.Ch15.

(14) US Census Bureau. Annual Estimates of the Resident Population for the United States, Regions, States, and Puerto Rico: April 1, 2010 to July 1, 2019 (NST-EST2019-01) https://www2.census.gov/programs-surveys/popest/tables/2010-2019/state/totals/nst-est2019-01.xlsx (accessed Jun 14, 2021).

(15) US Census Bureau. American Housing Survey https://www.census.gov/programs-surveys/ahs/data/interactive/ahstablecreator.html (accessed Jun 15, 2021).

(16) US Census Bureau. Historical Census of Housing Tables: Sewage Disposal https://www.census.gov/data/tables/time-series/dec/coh-sewage.html (accessed Jun 11, 2021).

(17) Weller, Z. D.; Hamburg, S. P.; von Fischer, J. C. A National Estimate of Methane Leakage from Pipeline Mains in Natural Gas Local Distribution Systems. *Environ. Sci. Technol.* **2020**, *54* (14), 8958–8967. https://doi.org/10.1021/acs.est.0c00437.

(18) Fischer, M. L.; Chan, W. R.; Delp, W.; Jeong, S.; Rapp, V.; Zhu, Z. An Estimate of Natural Gas Methane Emissions from California Homes. *Environ. Sci. Technol.* **2018**, *52* (17), 10205–10213. https://doi.org/10.1021/acs.est.8b03217.

(19) Gómez, D. R.; Watterson, J. D.; Americano, B. B.; Ha, C.; Marland, G.; Matsika, E.; Namayanga, L. N.; Osman-Elasha, B.; Saka, J. D. K.; Treanton, K. Stationary Combustion. In *2006 IPCC Guidelines for National Greenhouse Gas Inventories. Volume 2: Energy*; Eggleston, S., Buendia, L., Miwa, K., Ngara, T., Tanabe, K., Eds.; Institute for Global Environmental Strategies (IGES): Hayama, Japan, 2006; pp 2.2-2.47. https://doi.org/10.1007/BF00914340.

(20) Hajny, K. D.; Salmon, O. E.; Rudek, J.; Lyon, D. R.; Stuff, A. A.; Stirm, B. H.; Kaeser, R.; Floerchinger, C. R.; Conley, S.; Smith, M. L.; Shepson, P. B. Observations of Methane Emissions from Natural Gas-Fired Power Plants. *Environ. Sci. Technol.* **2019**, *53* (15), 8976–8984. https://doi.org/10.1021/acs.est.9b01875.

(21) EPA. Greenhouse Gas Reporting Program https://ghgdata.epa.gov (accessed Apr 16, 2021).

(22) EPA. Landfill Methane Outreach Program https://www.epa.gov/lmop/landfill-technical-data (accessed Apr 18, 2021).

(23) EPA. Clean Watersheds Needs Survey https://ordspub.epa.gov/ords/cwns2012/f?p=241:25: (accessed Jun 8, 2021).

(24) European Commission Joint Research Centre (JRC)/Netherlands Environmental Assessment Agency (PBL). Emission Database for Global Atmospheric Research (EDGAR), release version 4.2 https://edgar.jrc.ec.europa.eu/overview.php?v=42 (accessed Oct 2, 2020).

(25) Crippa, M.; Solazzo, E.; Huang, G.; Guizzardi, D.; Koffi, E.; Muntean, M.; Schieberle, C.;

Friedrich, R.; Janssens-Maenhout, G. High Resolution Temporal Profiles in the Emissions Database for Global Atmospheric Research. *Sci. Data* **2020**, *7* (1), 121. https://doi.org/10.1038/s41597-020-0462-2.

(26) European Commission Joint Research Centre (JRC)/Netherlands Environmental Assessment Agency (PBL). Emission Database for Global Atmospheric Research (EDGAR), release version 5.0 https://edgar.jrc.ec.europa.eu/overview.php?v=50_GHG (accessed Oct 2, 2020).

(27) Crosson, E. R. A Cavity Ring-down Analyzer for Measuring Atmospheric Levels of Methane, Carbon Dioxide, and Water Vapor. *Appl. Phys. B* **2008**, *92* (3), 403–408. https://doi.org/10.1007/s00340-008-3135-y.

(28) Dlugokencky, E. J.; Myers, R. C.; Lang, P. M.; Masarie, K. A.; Crotwell, A. M.; Thoning, K. W.; Hall, B. D.; Elkins, J. W.; Steele, L. P. Conversion of NOAA Atmospheric Dry Air CH4 Mole Fractions to a Gravimetrically Prepared Standard Scale. *J. Geophys. Res.* **2005**, *110* (D18), D18306. https://doi.org/10.1029/2005JD006035.

(29) Kantha, L. H.; Clayson, C. A. *Small Scale Processes in Geophysical Fluid Flows*; Academic Press: San Diego, 2000.

(30) Hanna, S. R. Applications in Air Pollution Modelling. In *Atmospheric turbulence and air pollution modelling*; Nieuwstadt, F. T., Van Dop, H., Eds.; Reidel: Dordrecht, The Netherlands, 1982; pp 275–310.

(31) Gately, C.; Hutyra, L. R. Anthropogenic Carbon Emission System, 2012-2017, Version 2. https://doi.org/10.3334/ORNLDAAC/1943.

(32) Gurney, K. R.; Liang, J.; Patarasuk, R.; Song, Y.; Huang, J.; Roest., G. Vulcan: High-Resolution Annual Fossil Fuel CO2 Emissions in USA, 2010-2015, Version 3. https://doi.org/10.3334/ORNLDAAC/1741.

(33) Gurney, K. R.; Liang, J.; Patarasuk, R.; Song, Y.; Huang, J.; Roest., G. Vulcan: High-Resolution Hourly Fossil Fuel CO2 Emissions in USA, 2010-2015, Version 3. https://doi.org/10.3334/ORNLDAAC/1810.
